# Loss-of-function Gα_s_ rare disease variants exert mutation-specific effects on GPCR signaling

**DOI:** 10.1126/scisignal.ado7543

**Published:** 2025-05-20

**Authors:** Theo Redfern-Nichols, Shannon L O'Brien, Xianglin Huang, Brian Medel-Lacruz, Davide Calebiro, Jana Selent, Graham Ladds, Maria Marti-Solano

**Affiliations:** 1 Department of Pharmacology, University of Cambridge, Tennis Court Road, Cambridge CB2 1PD, UK; 2 Department of Metabolism and Systems Science, College of Medicine and Health, University of Birmingham, Birmingham B15 2TT, UK; Centre of Membrane Proteins and Receptors (COMPARE), Universities of Nottingham and Birmingham, Birmingham B15 2TT, UK; 3 Research Program on Biomedical Informatics, Hospital del Mar Medical Research Institute, Department of Experimental and Health Sciences, Pompeu Fabra University, Barcelona, 08003, Spain

## Abstract

G protein–coupled receptors (GPCRs) are transmembrane detectors of extracellular signals that activate heterotrimeric G proteins to regulate intracellular responses. Because there are only 16 Gα proteins that can couple to GPCRs, variation in a single Gα can affect the function of numerous receptors. Here, we investigated two mutant forms of Gα_s_ (L388R and E392K) that are associated with pseudohypoparathyroidism type Ic (PHPIc), a maternally inherited rare disease. Gα_s_ is encoded by an imprinted gene, resulting in the mutant form of Gα_s_ being the only version of the protein present in certain tissues, which leads to tissue-specific disease manifestations. By integrating data from 3D structures, GPCR-G protein coupling specificity, transcriptomics, biophysics, and molecular dynamics with systems pharmacology modeling, we identified GPCRs whose signaling could be altered by Gα_s_ mutations in the kidney, a tissue involved in the pathophysiology of PHPIc. Analysis of G protein activation by the parathyroid hormone receptor 1 (PTH1R) revealed that L388R impaired Gα_s_ interaction with the receptor, whereas E392K reduced the receptor-induced activation of heterotrimeric G_s_. This indicates that different signal transduction steps can be altered by specific Gα_s_ mutants associated with the same disease. These findings highlight the importance of investigating mutation-specific perturbations in GPCR signaling to suggest patient-specific treatment strategies. Furthermore, our methods provide a blueprint for interrogating GPCR signaling diversity in different physiological and pathophysiological contexts.

## Introduction

G protein–coupled receptor (GPCR) signaling modulates key physiological processes such as vision, olfaction, neurotransmission, and metabolism. In this signaling system, hundreds of receptors have diversified to detect a wide collection of structurally-diverse signals that they relay to four different heterotrimeric G protein families, classified according to the type of Gα subunit they contain, to elicit an array of intracellular responses. Since the landmark publication of the structure of a prototypical ternary complex involving the β_2_ adrenergic receptor bound to a G_s_ heterotrimer in 2011 (1), the GPCR signaling field has greatly advanced on the concerted effort to understand receptor-G protein coupling from multiple dimensions. Information on the structure of GPCRs in complex with diverse G proteins is extensive (2), and systematic analyses of the functional coupling preferences of hundreds of receptors towards different Gα proteins have started uncovering the pairing rules governing GPCR signaling (3–5). Parallel developments in RNA sequencing and proteomics now allow us to assess how different signaling partners combine in specific cells, tissues, or organisms (6), thus giving rise to context-specific responses to the same stimulus (7). Taken together, all these insights have boosted our knowledge of GPCR function and offer the opportunity to address new holistic questions on receptor signaling.

Here, we have leveraged state-of-the-art multidimensional data on GPCR-Gα protein coupling to perform a systems pharmacology analysis of single-residue substitution mutations in the prototypical Gα_s_ protein that cause a rare disease. These substitutions (L388R and E392K), which have been detected in patients diagnosed with the endocrine disease pseudohypoparathyroidism type Ic (PHPIc) (8), provide a unique case study to address key questions on the context-specific effects of structural variation on GPCR signaling pathways. Although the L388R and E392K Gα_s_ mutants are capable of stimulating adenylyl cyclase after inhibition of GTP hydrolysis by cholera toxin, they display a loss of function with regards to their activation by GPCRs (8). Considering these residues are located in the helix 5 (H5) of Gα_s_, a key region determining the coupling and selectivity of G proteins towards GPCRs (9), both mutant forms have been postulated to impair Gα_s_-receptor interaction. Because the gene encoding Gα_s_ (*GNAS*) is in an imprinted locus, Gα_s_ transcripts show a highly specific pattern of expression. This results in Gα_s_ encoded by the maternal allele, which carries the mutations found in patients, being the only version of the protein that is present in some regions of the kidney, the thyroid, or the ovary, thus resulting in tissue-specific disease manifestations (10).

Our structural and network biology analyses revealed that L388R and E392K mutations have the potential to structurally and functionally impact Gα_s_ protein coupling to multiple receptors. By exploiting transcriptomics data to systematically evaluate which of these receptors are produced in proximal renal tubules, where the main rare disease phenotype occurs, our results identify particular GPCRs which, together with the parathyroid hormone receptor 1 (PTH1R), could display compromised signaling in patients. Using a detailed characterization of both Gα_s_ mutants at multiple signal transduction steps using state-of-the-art biophysical methods, we uncovered mutation-specific functional impairment, which we further explored through systems pharmacology modelling and molecular dynamics studies. Our models also enable the identification of potential patient-tailored treatment strategies based on individual rare disease variants and the prediction of how tissues lacking imprinting could be affected by Gα_s_ heterozygosity. In summary, this study exemplifies how obtaining a multidimensional view of GPCR signaling allows for the dissection of receptor-G protein signal transduction and the prediction of the functional impact of structural variation in GPCR pathway components from a context-based perspective. This, in turn, can foster a patient-specific understanding of phenotypic variation and disease and inspire new strategies for personalized treatment.

## Results

### Structural, functional, and tissue-specific Gα_s_ coupling analysis

**Fig. 1:**
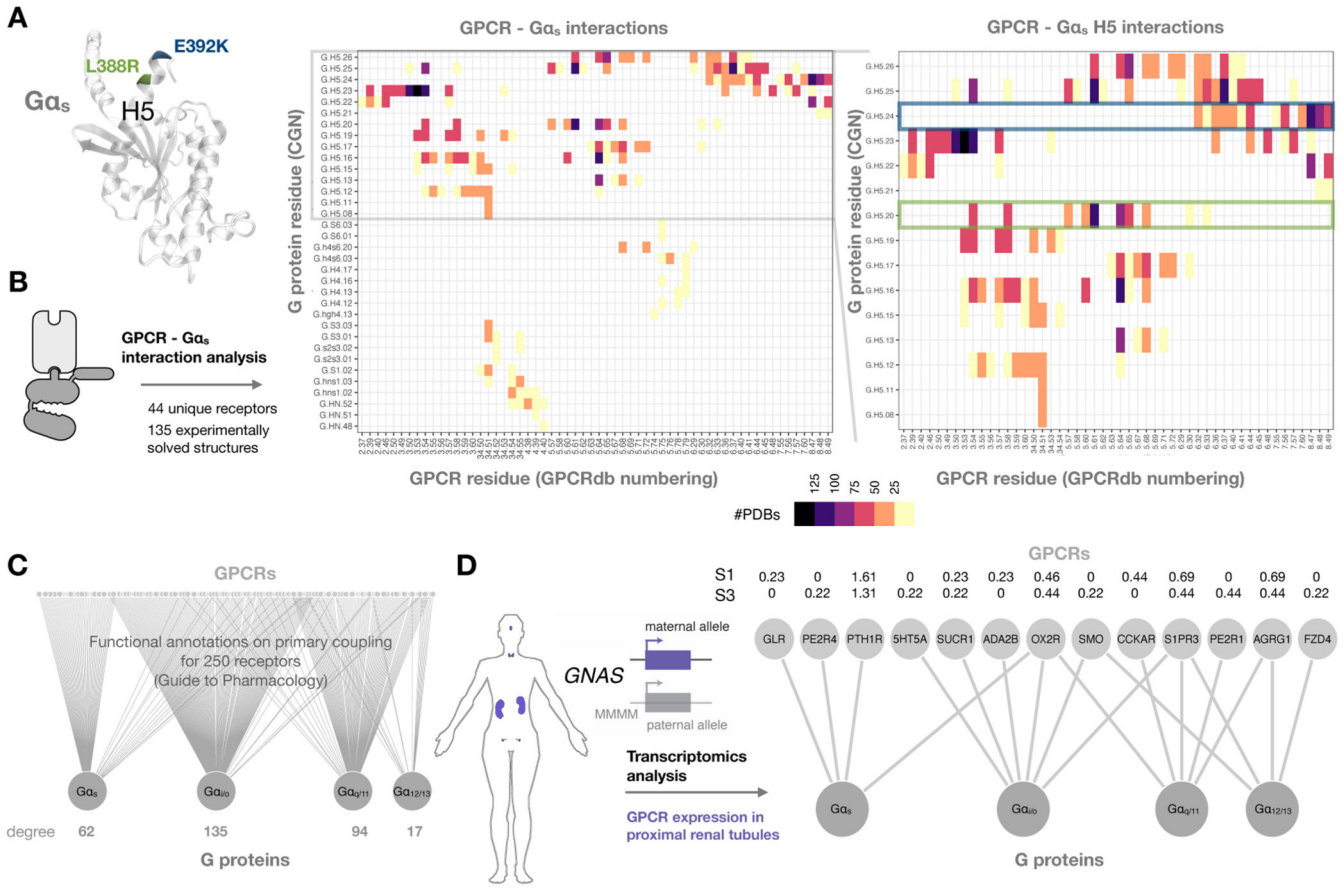
A context-based analysis of Gα_s_ coupling. (**A**) Structural mapping of rare disease variants L388R and E392K into an activated Gα_s_ structure (PDB: 6NBF). Both are located in the helix 5 (H5) of the Gα_s_ subunit. (**B**) Interaction analysis of 135 experimentally solved GPCR-Gα_s_ structures. Interactions present in at least 10 independent structures are shown in two heatmaps with residues annotated using GPCRdb generic numbering for receptors and the G protein common numbering (CGN) scheme for Gα_s_. Heatmaps show all GPCR interactions with Gα_s_ and interactions specifically between the receptor and Gα_s_ H5. Interactions of Leu^388^ (Leu^G.H5.20^) and Glu^392^ (Glu^G.H5.24^) are highlighted in green and blue boxes, respectively. (**C**) Bipartite graph of primary couplings between GPCRs and Gα proteins as annotated in the Guide to Pharmacology. Degree values indicate the number of GPCRs that primarily couple to a particular Gα family. (**D**) Schematic representation of tissues displaying maternal monoallelic expression from the *GNAS* locus due to imprinting and filtered bipartite graph of primary couplings between GPCRs and Gα protein families for receptors encoded by transcripts expressed in proximal renal tubules (16) with normalized expression amounts shown for S1 and S3 renal sub-segments.

To gauge the structural relevance of the mutations L388R and E392K on Gα_s_, we first mapped these positions into an active Gα_s_ protein structure (11) (PDB: 6NBF), which showed both residues are located in the H5 region of the Gα_s_ subunit (Fig. 1A). We next performed an analysis of all structurally solved GPCR-Gα_s_ complexes to quantitatively assess the contribution of these residues towards receptor-Gα protein interactions in a comprehensive manner. To do so, we analyzed contacts between Gα_s_ and receptors from 135 experimentally solved crystal and cryogenic electron microscopy (cryo-EM) structures, including 44 unique GPCRs (data file S1). Assigning each receptor and G protein residue a GPCRdb (12) and Common G Protein (CGN) (13) numbering scheme identifier, respectively, allowed us to obtain residue contact frequencies across all available structures (Fig. 1B). A general overview of these contact frequencies confirms previous observations on the overall importance of the Gα_s_ H5 in establishing an active GPCR-G protein interface (Fig. 1B, left). By focusing on this specific region, we observed how disease-related residues Leu^388^ and Glu^392^, corresponding to CGN G.H5.20 and G.H5.24 (Fig. 1B, right), establish frequent and extensive contacts with receptors. In the case of Leu^388^ (Leu^G.H5.20^), interaction occurs with transmembrane (TM) helices 3, 5, and 6 of GPCRs, with contacts between this residue and receptor positions 5.61 and 5.64 found in 102 and 96 out of the 135 analyzed structures, respectively. Glu^392^ (Glu^G.H5.24^) contacts are established with TM6 and TM7 of receptors, as well as receptor helix 8. This Gα_s_ residue shows a high contact frequency with this last helix, with interactions with positions 8.47 and 8.48 found in 109 and 97 analyzed structures, respectively. Taken together, this analysis highlights the potential generalized effect of mutations in these Gα_s_ positions when it comes to coupling to an array of GPCRs.

To further assess the extent of this effect, we performed a systematic analysis of GPCR signaling preferences for the four different Gα protein families. We retrieved information on primary receptor couplings available in the GproteinDb (14) and originally annotated in the Guide to Pharmacology (15) to generate a GPCR-Gα functional coupling graph (Fig. 1C). This revealed that the function of at least 62 different GPCRs, which preferentially couple to Gα_s_, could be theoretically affected by the rare disease mutations. We know, however, that considering tissue-specific context is highly relevant in the rare disease due to the fact that the *GNAS* locus is imprinted in a number of tissues, leading to the production of a single maternally-inherited version of Gα_s_ that is mutated in patients (8). This is particularly relevant in the proximal renal tubules of the kidney, where the disease results in disrupted calcium resorption. For this reason, we mined publicly available transcriptomics data from different human kidney segments (16) to filter the initial GPCR-Gα network so that it only included those receptors expressed in the proximal renal tubules (Fig. 1D). This revealed that the most highly expressed transcript encoding a receptor displaying primary Gα_s_ coupling in this tissue was the parathyroid hormone receptor 1 (PTH1R), in line with previous observations on the role of this GPCR in the aetiology of the rare disease (8). Transcriptomics analysis also showed that other receptors capable of coupling to Gα_s_, like the glucagon receptor (GLR), prostaglandin E2 receptor 4 (PE2R4) and orexin receptor type 2 (OX2R), as well as receptors with reported secondary couplings to Gα_s_ (α2B-adrenoceptor (ADA2B) and cholecystokinin-1 receptor (CCKAR)), could be present in the tissue, opening new questions on their potential role in PHPIc.

### Rare disease variants alter distinct signal transduction steps

**Fig. 2:**
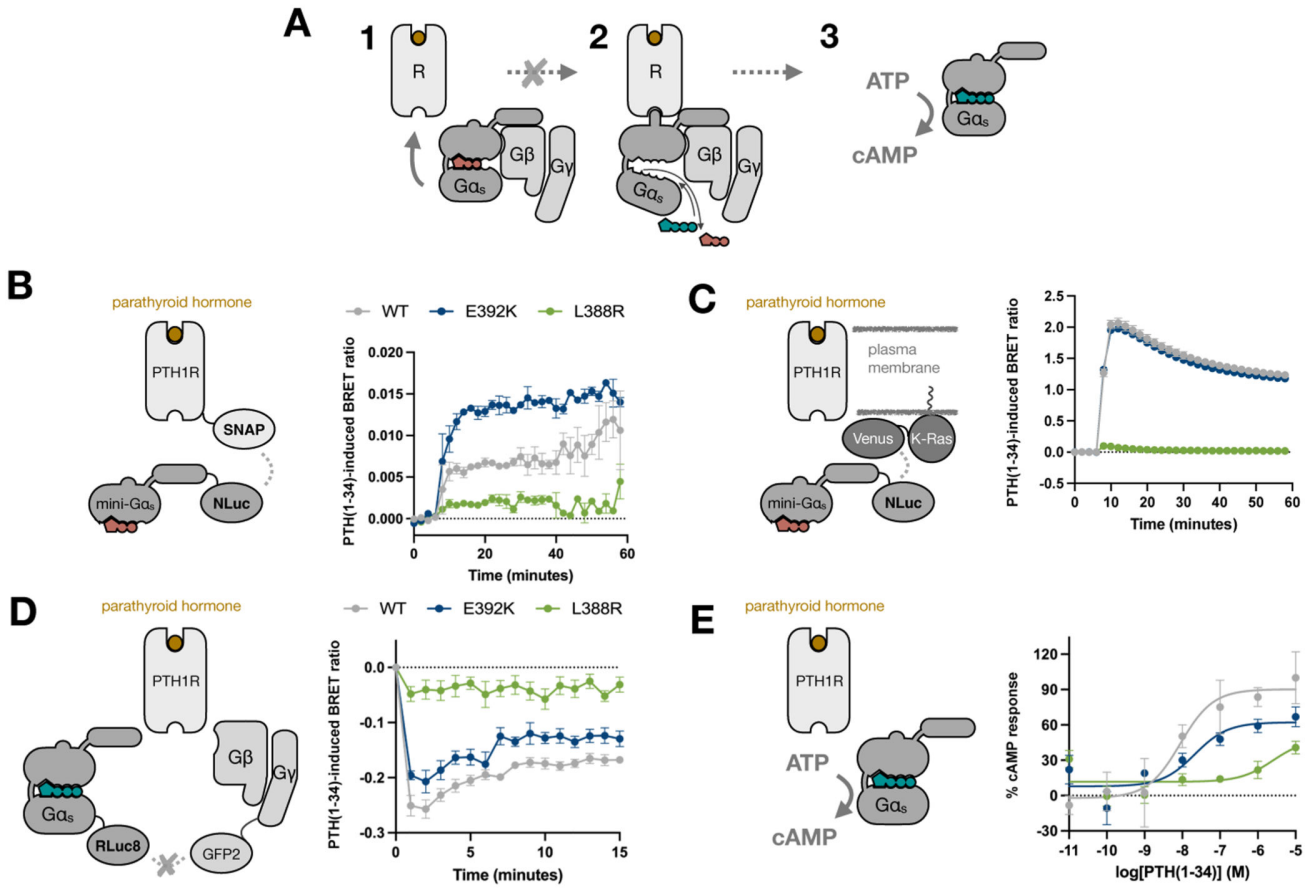
Monitoring differential receptor engagement in Gα_s_ rare disease variants. (**A**) Schematic representation of key steps in the G protein activation process, including heterotrimeric G protein coupling to an activated receptor (R) (step 1), receptor-induced exchange of GDP (ochre) for GTP (teal) (step 2), and Gα_s_-GTP–mediated cAMP production after dissociation from the Gβγ subunits (step 3). The grey cross represents the activation step reported to be compromised upon L388R or E392K mutation. **(B and C)** BRET assays used to measure mini-Gα_s_ (WT, E392K, or L388R) translocation to PTH1R (B) or to the plasma membrane marker K-Ras (C) following stimulation with 1μM PTH (1-34). The BRET ratio was calculated as acceptor/donor wavelength and baseline-corrected with the value obtained with vehicle control. (**D**) BRET assays to measure the dissociation of heterotrimeric G_s_ containing WT, E392K, or L388R Gα_s_, as determined using the TRUPATH G protein activation assay following stimulation of the PTH1R using 1μM PTH(1-34). The BRET ratio was calculated as acceptor/donor wavelength and baseline-corrected with the value obtained with DMSO control. (**E**) BRET assays to measure the differential cAMP accumulation induced by WT or mutant Gα_s_ upon PTH1R stimulation. cAMP accumulation after 1hr stimulation with 10μM-0.01nM PTH(1-34) was measured using the LANCE cAMP kit in HEK293ΔGNAS cells transiently transfected with Gα_s_ WT, E392K, or L388R. The data were normalized to the maximum cAMP production observed for WT Gαs. All values are represented as mean ± SEM, with n=3 independent experimental repeats in duplicates.

Initial work characterizing L388R and E392K mutations concluded these rare disease variants were likely to disrupt the receptor signaling process by preventing GPCR-Gα protein interaction (8) (Fig. 2A). To dissect their influence on PTH1R signal transduction processes, we first took advantage of mini-G proteins – engineered variants of the GTPase domains of Gα subunits (17) – to directly monitor Gα_s_ recruitment to PTH1R excluding any interference from receptor coupling to additional Gα proteins. Through the use of mini-Gα_s_ proteins bearing each mutation, we were able to measure their interaction with PTH1R, as well as their recruitment to different membrane compartments, upon activation by parathyroid hormone 1-34 (PTH(1-34)) using bioluminescence resonance energy transfer (BRET). As expected, the L388R mutant showed impaired receptor recruitment upon agonist stimulation, which was also reflected by a diminished translocation to the plasma membrane marker, K-Ras (Fig. 2, B and C, green curve). This is also in line with previous work (18) monitoring recruitment of L388R Gα_s_ to the melanocortin 4 and growth hormone–releasing hormone receptors using a NanoBIT complementation assay, showing this mutant exhibits an impaired receptor interaction. The E392K mutant showed a completely different behavior, displaying higher amounts of receptor interaction than did the non-mutated mini-Gα_s_ despite showing comparable plasma membrane interaction (Fig. 2, B and C, blue curve). The contrasting effects of both mutants on receptor interaction could be also observed at endosomal compartments (fig. S1). Agonist-dependent recruitment of L388R to endosomal membranes was also lost, whereas E392K mini-Gα_s_ was still recruited to endosomes, albeit at a lower degree than non-mutated mini-Gα_s_. This would be in line with observations highlighting the capacity of mini-G proteins to inhibit receptor internalization in a way that reflects Gα binding preferences (19).

To further investigate these differences, we monitored a subsequent step in receptor-mediated G protein activation by measuring the ligand-induced dissociation of the heterotrimeric G_s_ protein complex. To do so, we followed the loss of BRET signal between Gα_s_ and Gβγ upon PTH1R stimulation with 1 μM PTH(1-34) using the TRUPATH platform (20). Consistent with other examples using TRUPATH to study Gα_s_ activation, following stimulation with PTH(1-34), the wild-type G protein displayed a rapid loss of BRET signal, indicative of dissociation between Gα_s_ and Gβγ followed by a gradual rise to steady state. In line with our observations showing impaired L388R interaction with PTH1R, a Gα_s_ version including this mutation displayed minimal ligand-induced G protein dissociation (Fig. 2D). Crucially, and in accordance with past observations (8), this was not due to a reduction in protein amounts upon introduction of the mutation (fig. S2A). The E392K mutant, conversely, showed an intermediate response, with ligand-induced Gα_s_-Gβγ dissociation being significantly lower than in the wild-type G_s_ protein (as measured by area under the curve (AUC) *p* = 0.0014; *n* = 6, fig. S2B). These results are in accordance with previous observations for the downstream responses with these mutants (8), where E392K and L388R display intermediate and more pronounced, respectively, loss of function with regards to cyclic adenosine monophosphate (cAMP) accumulation (Fig. 2E, 67% and 41% of the wild-type at the highest PTH(1-34) concentration). These differences were also reproduced by using the TRUPATH platform to monitor isoprenaline-induced G_s_ protein activation at the prototypical β2-adrenoceptor (fig. S2C). Overall, these analyses highlight how distinct mutations leading to the same rare disease can do so by disrupting distinct steps in the signal transduction process.

### A systems pharmacology model for impaired G protein function

**Fig. 3:**
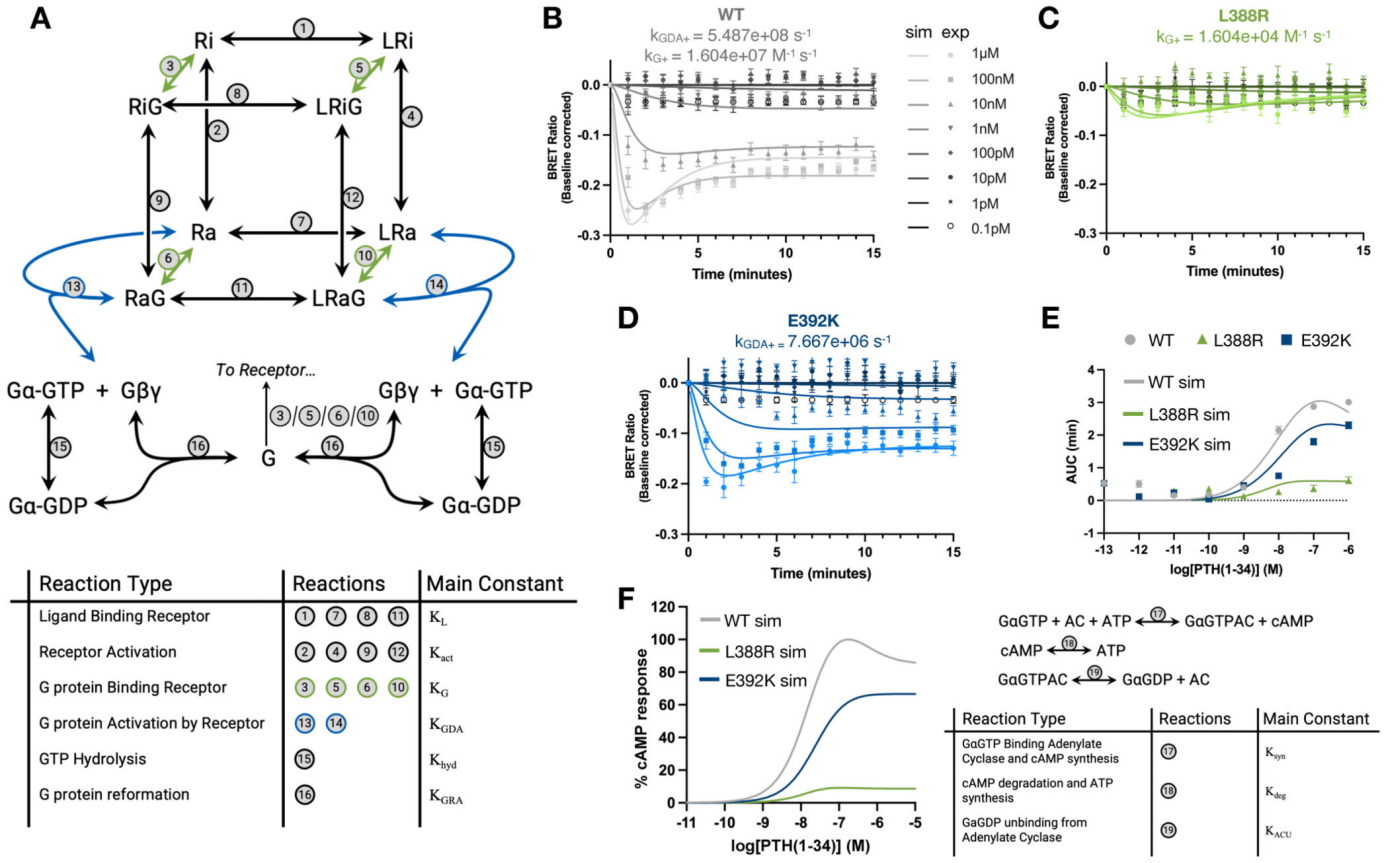
A systems pharmacology model of Gα_s_ activation. (**A**) Cubic ternary complex model of GPCR signaling with extended reactions enabling simulation of the G protein activation cycle. Reactions are summarized in the table with associated equilibrium constants. Reactions using the same parameters to simulate all three Gα_s_ variants (WT, E392K, and L388R) are shown in grey. Reactions that have been changed to simulate variant effects are shown in green for L388R and in blue for E392K. (**B to D**) Experimental (exp) time-course values for the TRUPATH G protein activation assay following 15-minute stimulation of the PTH1R using 1μM – 0.1pM PTH(1-34) for WT (B), L388R (C), E392K (D) are represented as points. Simulated (sim) values are represented by solid lines fitted using the model shown in (A) with the parameter changes indicated in each panel. (**E**) Experimental (exp) concentration-response values for WT Gα_s_ and Gα_s_ containing the L388R or E392K mutation, measured as the area between the kinetic trace and baseline for each concentration (0.1pM-1μM of PTH(1-34)), are represented as points. Solid lines represent simulations (sim) for each Gα_s_ protein spanning the 0.1pM to 1μM concentration range. (**F**) Simulated concentration-response curves of cAMP production (measured at peak cAMP) after stimulation with 0.1pM to 1μM PTH(1-34). The right panel shows the additional simplified reactions appended to the model in (A) enabling simulation of cAMP production. For all panels, experimental values were obtained from n=3 independent experimental repeats in duplicates.

The observed diversity in mutant behavior prompted us to further explore which specific steps in the receptor-mediated G protein activation cycle were affected by the different rare disease variants of Gα_s_. To understand the basis for these observations, we generated a systems pharmacology model of G protein activation using ordinary differential equations (Fig. 3A, tables S1 and S2). Initially, the model was fitted to the data points from the TRUPATH experiments for the wild-type Gα_s_ (Fig. 3B) using optimization algorithms to minimize the squared error between simulation and experimental data as described previously (21). Next, we systematically explored perturbations in model parameters that could explain the G protein activation differences measured in the two Gα_s_ mutants (fig. S3). Based on our observations of differential mini-Gα_s_ recruitment to PTH1R (Fig. 2B), we inferred that, for the L388R mutant, G protein binding to the agonist-stimulated GPCR was impaired. This behavior was reproduced in our model by reducing k_G+_, the constant governing G protein binding to different receptor activation states, by 1000-fold (k_G+_ of 1.604x10^7^ M^-1^s^-1^ for the wild-type G protein vs. 1.604 x10^4^ M^-1^s^-1^ for L388R) (Fig. 3C and table S3). For the E392K mutant, the observed reduction in the Gα_s_-Gβγ dissociation was replicated by decreasing the parameter describing the capacity of the G protein to be activated by a GPCR (k_GDA_) by 72-fold compared to the wild type (k_GDA+_ of 5.487x10^8^ s^-1^ vs 7.667x10^6^ s^-1^) (Fig. 3D and table S3). These parameter changes allow our simulated curves to closely fit the kinetic data from the original TRUPATH experiments (Fig. 3E).

Furthermore, expanding our newly created model to estimate cAMP production by the wild-type and mutated Gα_s_ variants reproduced our previously characterized signaling trends (Fig. 2E), which were also observed when the L388R and E392K mutations were originally characterized (8). In this way, this expanded model replicates the conservation of ligand-induced half-maximal effective concentration (EC50) for the L388R and E392K mutants, together with the previously observed loss-of-function pattern for cAMP response at the highest PTH(1-34) concentration (Fig. 3F, 68% and 10%, respectively, compared to the wild type). Collectively, insights from our systems pharmacology modeling further illuminate how the L388R mutant could present a loss of function related to impaired receptor association, whereas the E392K mutation could lead to a suboptimal receptor-mediated activation of Gα_s_.

### Mutations alter GPCR-G protein interfaces and dynamics

**Fig. 4:**
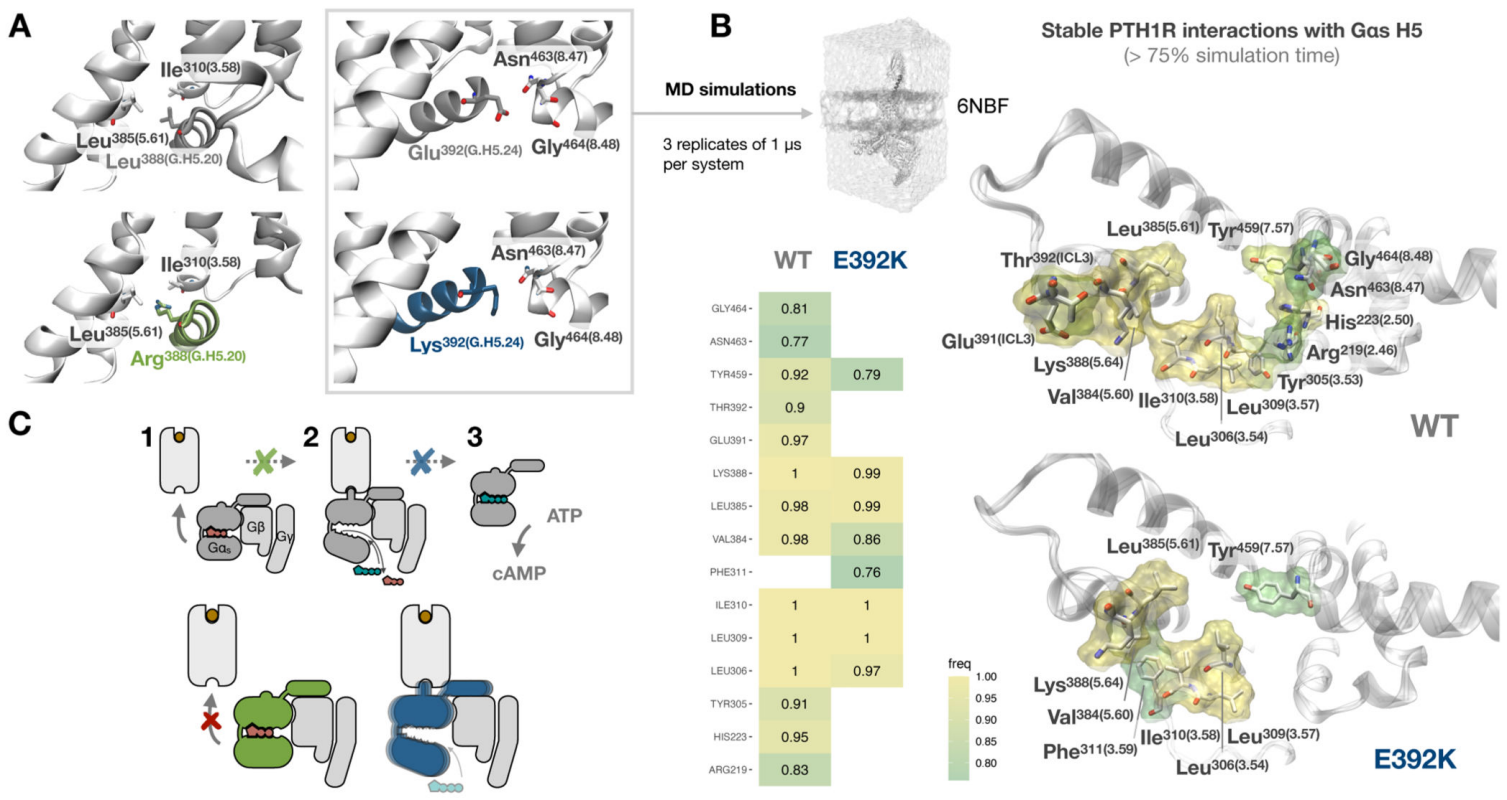
Structural analysis of PTH1R-Gα_s_ interactions. (**A**) Structural model of mutation effects based on the structure of PTH1R bound to Gα_s_ and a PTH analogue (PDB: 6NBF). The upper panels show Leu^388^ and Glu^392^ (Leu^G.H5.20^ and Glu^G.H5.24^, respectively) interactions between wild-type Gα_s_ protein (grey) and PTH1R receptor residues (white). The lower panels show a model of the potential effects of L388R (green) and E392K (blue) mutations on receptor interaction. Residues have been annotated using GPCRdb generic numbering for receptors and the G protein common numbering (CGN) scheme for Gα_s_. (**B**) Analysis of stable interactions of the PTH1R receptor with Gα_s_ H5 during molecular dynamics (MD) simulations. Receptor residues establishing stable interactions (measured as residues that interact >75% of the simulation time across 3 independent MD replicates) are shown as sticks in an intracellular view of the receptor structure (PDB: 6NBF) and colored according to interaction frequency (freq). The structural representation includes frequencies for the simulations including wild-type (WT) and mutated (E392K) Gα_s_ proteins. Specific frequency values for both systems are presented in the heatmap. (**C**) Schematic model of mutation-specific coupling deficiencies. The L388R mutant (green) could present defective interactions with activated GPCRs (disrupting step 1 in the scheme), and the E392K mutant (blue) could lead to impaired receptor-mediated G protein activation (disrupting step 2).

To gain a structural understanding of the potential effects of these rare disease mutations on GPCR-Gα_s_ interactions, we took advantage of the cryo-EM structure of PTH1R bound to Gα_s_ and a PTH analogue (11) (PDB: 6NBF). Modelling each mutation into the solved Gα_s_ structure clearly suggested why one of the mutations may have a more pronounced effect on GPCR-Gα_s_ complex formation: Specifically, mutating a leucine to an arginine at position 388 of Gα_s_ (G.H5.20 following the CGN), would replace a set of hydrophobic contacts with adjacent residues Ile^320^ and Leu^385^ (Ile^3.58^ and Leu^5.61^ following GPCRdb numbering, respectively) in the receptor interface with a larger, positively charged residue, thus generating a highly unfavorable interaction and potentially an intermolecular clash (Fig. 4A). Therefore, upon L388R mutation, GPCR-Gα_s_ complexes could display substantial conformational rearrangements as compared to wild type. In contrast, although mutation from a glutamic acid into a lysine could modify interactions established by the wild-type Gα_s_ with the backbone of residues Asn^463^ and Gly^464^ (Asn^8.47^ and Gly^8.48^, respectively) in the receptor, this mutation could still be accommodated and allow for alternative interactions making it more complex to evaluate its functional impact by analyzing the structural model.

The relatively subtle structural effect observed for the E392K mutation as compared to the wild-type Gα_s_ prompted us to undertake a more detailed comparison of these two Gα_s_ proteins by assessing their stability in complex with PTH1R using classic molecular dynamics (MD) simulations. Although the Gα_s_ H5 maintained its helical conformation in both the wild-type protein and the E392K mutant across 3 independent replicates of 1 μs (fig. S4), analysis of stable contacts (occurring during >75% of the simulation time) between H5 and the receptor revealed a decrease in the number of contacts and in their frequency for the E392K mutant as compared to the wild-type Gα_s_ (Fig. 4B, see data file S2 for a list of all interactions). Mapping these contact frequencies into the receptor intracellular interface showed how, although the E392K mutant preserves crucial stable interactions with residues in TM domains 3, 5 and 7; it lacks key contacts with TM2, the intracellular loop 3 (ICL3), and H8 that are established by the wild-type Gαs. This incomplete engagement of H5 in the nucleotide-free state of the G protein may explain why the receptor-mediated Gα_s_ transition into a fully activated GTP-bound state is partially impaired upon E392K mutation. Altogether, our structural analyses are in line with our previous experimental observations and support a mechanistic model in which one of the mutations, L388R, would result in Gα_s_ losing its signaling capacity due to a lack of interaction with GPCRs (Fig. 4C, green), whereas the other, E392K, could be a product of GPCR–Gα_s_ complex instability, resulting in a partial stalling of the G protein activation process (Fig. 4C, blue).

### Variant-specific drug responses and cell signaling diversity

**Fig. 5:**
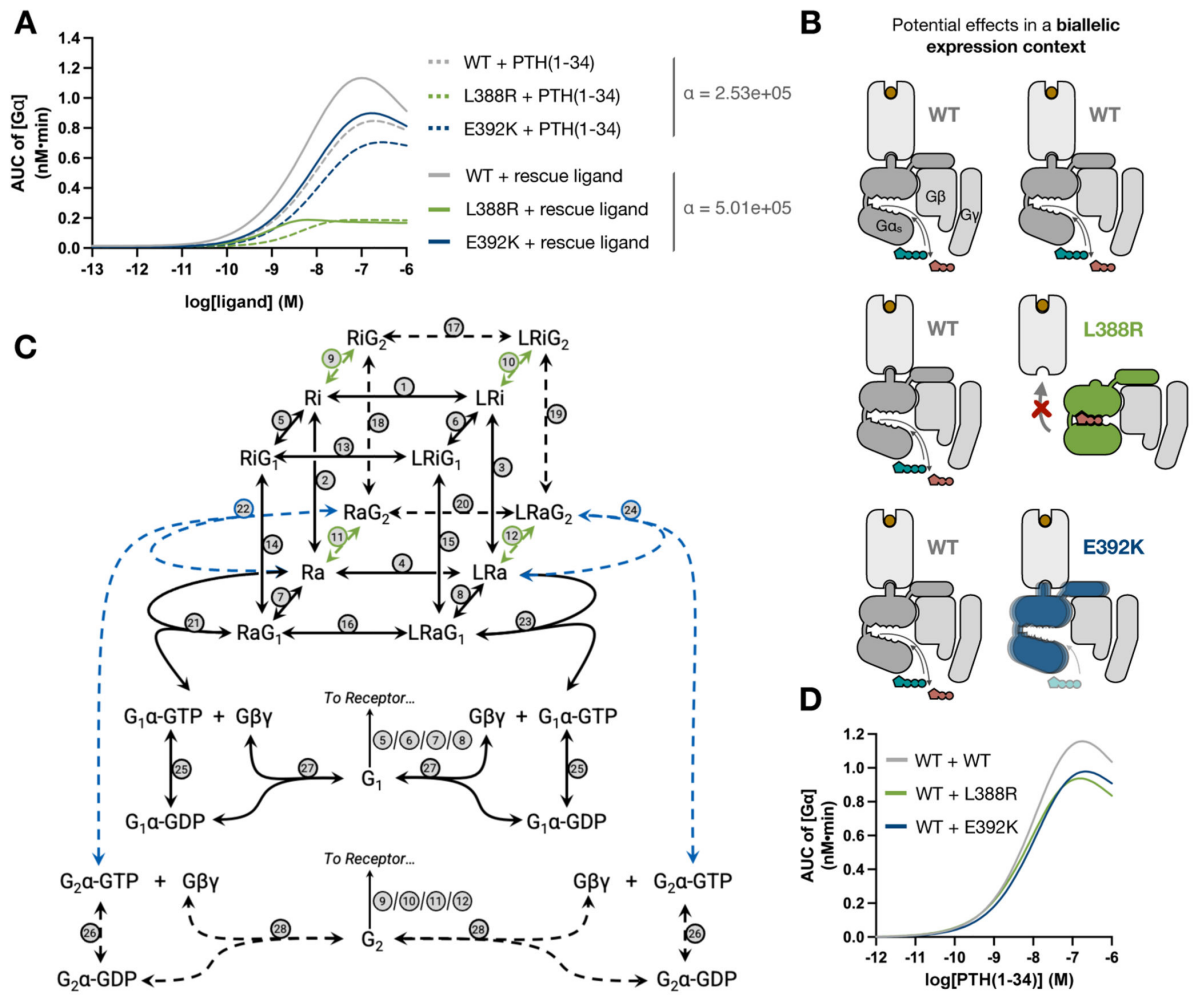
Modeling mutation rescue strategies and heterozygosity effects. (**A**) Simulation of ligand effects based on the cubic ternary complex model of GPCR signaling (Fig. 3A). Original curves corresponding to total WT and mutant Gα accumulation in response to PTH(1-34) are shown as dashed lines, and signaling in response to a new theoretical rescue ligand with a two-fold change in ligand-receptor cooperativity (α) is shown as solid lines. (**B**) Schematic representation of expected Gα_s_ protein diversity in healthy individuals (top) and rare disease patients bearing the L388R (middle, green) or E392K (bottom, blue) heterozygous mutation in tissues where the *GNAS* locus is not imprinted and there is biallelic Gα_s_ expression. (**C**) Dual G protein cubic ternary complex model where the additional G protein can be WT, L388R, or E392K Gα_s_. Reactions that have been changed to simulate variant effects are shown in green for L388R and in blue for E392K. (**D**) Simulated curves for total free Gα from the dual G protein model using the reactions presented in Fig. 3.

The differences in individual mutant behavior observed in our multidimensional study open critical questions when it comes to suggesting new potential treatments that could rescue Gα_s_ signaling in disease-associated tissues. Even if both L388R and E392K mutations give rise to pseudohypoparathyroidism, successfully developing a potential ligand for restoring cAMP production upon PTH1R stimulation could be highly mutation-dependent. To explore the ideal theoretical properties of such a ligand, we employed our previously developed systems pharmacology model to explore ligand parameter space. Our simulations confirmed that altering ligand properties in the presence of the L388R mutation does not allow for rescue of G protein activation (fig. S5 A and B); conversely, a compound promoting a two-fold increase in ligand-receptor cooperativity (α) could rescue Gα_s_ activity in the E392K mutant (Fig. 5A). This could be achieved either by a rescue ligand with both faster association and slower dissociation with the active receptor or by individually altering one of these properties (fig. S5 C and D). Therefore, our prediction is consistent with previously suggested therapeutic approaches based on the use of long-acting PTH derivatives to treat hypoparathyroidism patients (22). In more general terms, this exercise exemplifies why characterizing specific causative mutations in individual patients can be critical when evaluating potential strategies for their treatment.

Another key aspect that can be explored with our models relates to the molecular phenotypes we can expect in nonimprinted tissues. In these tissues, patients will express both the wild-type paternal and loss-of-function maternal copies of *GNAS*, leading to the production of wild-type and mutated Gα_s_ in the same cell (Fig. 5B). To determine how the presence of two Gα_s_ versions with differing properties could alter overall signaling output, we built on our previous work simulating differential Gα_s_ coupling to the adenosine A1 receptor (21) to generate an extended model accounting for Gα_s_ heterozygosity (Fig. 5C and table S4). This model shows that, in comparison to a system including two copies of the wild-type Gα_s_, heterozygous production of both the wild-type protein and either the L388R or E392K variant would produce an equivalent amount of total free Gα_s_ in response to PTH1R stimulation, corresponding to 81.3% and 81.8% of the maximum response, respectively (Fig. 5D). In line with our previous mechanistic insights, this can be explained by a compensatory effect wherein the L388R mutant, which shows deficient GPCR binding, does not compete with the wild-type Gα_s_, allowing it to freely engage with activated receptors (fig. S6 A and B), whereas the E392K mutant would still contribute to Gα_s_ dissociation but with a lower efficacy than does the wild-type protein, thus interfering with the full activation of the pathway (fig. S6 A and C). The observation that overall loss in G protein activation in this model (~18%) is much lower than that observed in the presence of individual Gα_s_ mutants, may explain why phenotypes associated with PHPIc mutations are predominantly restricted to tissues in which only the maternally inherited, mutant allele of *GNAS* is expressed. These observations highlight the importance of accounting for heterozygosity when assessing variant effects present in the GPCR signaling machinery.

## Discussion

Here, we combined comparative structural analyses, network biology reconstruction, transcriptomics mining, biophysical characterization, systems pharmacology modeling, and molecular dynamics simulations to explore how rare disease variation translates into context-specific signaling impairment in the GPCR system. In particular, our results revealed why specific receptors like PTH1R are uniquely affected by rare disease mutations in a central signaling node such as the Gα_s_ protein. We also showed how L388R and E392K, two Gα_s_ mutations giving rise to the same pathological phenotype, can do so by altering different steps of the signal transduction process. Furthermore, our models provide a mechanistic explanation for the alterations in molecular interaction properties and intermolecular complex stability that could underlie defective signaling by each mutant. Our models also allow exploration of new theoretical strategies for mutation-specific signaling rescue and illustrate why monoallelic vs biallelic Gα_s_ expression can result in tissue-specific dysfunction in rare disease patients.

Although this study represents a paradigmatic example on the power of multidimensional analyses to understand receptor signaling variability, a deeper examination of downstream signaling pathways will be needed to fully characterize defective parathyroid hormone function in patients. This is due to the fact that additional signaling responses are mediated by PTH1R coupling to other intracellular partners like Gα_q/11_ proteins and β-arrestins (23), whose interaction properties with the receptor could be indirectly modified by changes in Gα_s_ engagement. Additionally, the analysis pipeline presented in this work could be extended to other imprinted tissues to clarify additional observations of hormonal resistance found in some PHP patients (10). These analyses would also benefit from an *ad hoc* exploration of receptor abundance, because some members of the GPCR family are known to be detected in low amounts in transcriptomics and proteomics datasets (24). Finally, although our modeling and molecular dynamics simulation studies offer a mechanistic explanation for the observed effects of Gα_s_ mutants on receptor signal transduction, further developments in molecular dynamics simulation capabilities will be needed to comprehensively simulate how the L388R mutation alters the process of Gα_s_ recognition by GPCRs. Furthermore, additional structural evidence on G protein coupling intermediates could shed critical light on the steps in receptor coupling that are disrupted with each mutant protein. In the case of Glu^392^, this could be particularly relevant considering that molecular dynamics studies (25) have highlighted its role in the first steps of Gα_s_ binding to an intermediate activation state of the β2-adrenoceptor. In this way, additional insights could be facilitated by the new time-resolved cryo-EM strategies that have been applied to explore Gα_s_ coupling to class A receptors (26) and could reveal the role of this mutated position in PTH1R recognition and nucleotide shedding.

Beyond our specific case study, this work can provide some fundamental guidance when it comes to characterizing functional variation in the GPCR signaling system. This can be the case whether variation originates from disease-causing mutations or from common polymorphisms present in the general population that could affect the predisposition to develop certain pathological phenotypes or to differentially respond to particular GPCR-targeting drugs in the clinic (27). First, our results exemplify the importance of monitoring orthogonal signaling outputs while characterizing a set of variants related to a specific phenotype, because each of our biophysical analyses in isolation would have led us to contrasting conclusions when assessing the gain or loss of function of individual mutants. Second, our work highlights the need to consider the heterozygous expression of different versions of the same GPCR pathway component while attempting to determine whether variation will result in significantly different signaling effects. In this sense, the models presented here can serve as a blueprint to inspire future systems pharmacology analyses incorporating these considerations. Finally, our insights exemplify how identifying individual causal mutations in specific patients could be critical to guide the choice of therapeutic strategies and maximize their chances of success, supporting ongoing initiatives to transition into new personalized medicine treatment frameworks.

## Materials and methods

### Gα_s_-receptor protein contact analysis

Structures for the following 135 receptors in complex with Gα_s_ were downloaded from the RCSB PDB (28) (https://www.rcsb.org): 6P9X, 7F4I, 7VUI, 7L1U, 6VN7, 7JOZ, 6WI9, 7CKY, 6P9Y, 7TZF, 7LCI, 7KI1, 7VUJ, 6XOX, 6VCB, 7CX3, 7RMG, 7CKW, 7DUR, 7E14, 7RG9, 7S3I, 7DHR, 7TYX, 6LI3, 7V35, 7BB6, 6ORV, 7D3S, 7F53, 7LJC, 7JVP, 7F58, 7XTC, 7PIV,7CFN, 7TYH, 7P02, 7WU3, 6E67, 7VAB, 6X1A, 6M1H, 7TYY, 7RMH, 7TYF, 7DUQ, 7PIU, 7F54, 7TYI, 7KI0, 7D7M, 7EVM, 7F4H, 7BB7, 7XT8, 6X18, 7CZ5, 5UZ7, 7BZ2, 6NIY, 7VBI, 7RGP, 7RBT, 7F4D, 5VAI, 6PB0, 7RA3, 7CX2, 6PB1, 6LPB, 7TYW, 6WPW, 7RTB, 7JV5, 7LLL, 7F16, 7DW9, 7XTB, 7TYO, 6LMK, 6WHC, 6WZG, 6GDG, 7DHI, 7CKZ, 7CKX, 7VUH, 6B3J, 7EVW, 7BW0, 7CRH, 7LJD, 7F55, 7L1V, 6M1I, 7AUE, 6E3Y, 7S1M, 5G53, 7DH5, 7LLY, 7RMI, 7TYL, 6X19, 6UUN, 7EZK, 6UUS, 7WUJ, 7MBX, 7VBH, 7CX4, 7CFM, 6NI3, 7F4F, 7V9M, 7WU2, 7KH0, 6UVA, 7TYN, 7C2E, 7JVQ, 6NBF, 7JJO, 7FIG, 7D68, 6NBI, 7DTY, 7FIY, 7FII, 3SN6, 7FIN, 7FIH, 7FIM, 6NBH. Contacts between each receptor-Gα_s_ protein pair were calculated using Arpeggio (29) with default parameters. Annotations regarding the G protein common numbering scheme were downloaded from the GproteinDb (14) resource and receptor residues were assigned generic numbering using the GPCRdb API (30). Our final analysis considered all receptor-Gα_s_ protein interactions that occurred in at least 10 independent structures (that is, structures from independent PDB entries, data file S1) and final interaction plots were obtained using RStudio 2022.07.2.

### Functional coupling and context-specific GPCR expression

Information on receptor couplings as annotated in the Guide to Pharmacology (15) was downloaded from GproteinDb (14) and primary G protein couplings were represented as a bipartite graph using the igraph R library. Transcriptomics data from the human kidney was obtained via the Kidney Transcriptomics Data resource from the Epithelial Systems Biology Laboratory at NHLBI, NIH, Bethesda, MD (https://esbl.nhlbi.nih.gov/Databases/KSBP2/Targets/TranscriptomicData.html). To obtain a tissue-specific bipartite graph we used the dataset obtained by Cheval *et al*. (16) which is publicly available at https://doi.org/10.1371/journal.pone.0046876.s003 to search for GPCRs expressed in the S1 and S3 sub-segments of the proximal renal tubule.

### Mini-Gα_s_ interactions with PTH1R and membrane markers

A plasmid encoding PTH1R-SNAP was kindly provided by Ulrike Zabel and K-Ras-Venus was provided by Kevin Pfleger, University of Western Australia (31). NanoLuc (NLuc) luciferase-tagged mini-G probes were kindly provided by Nevin Lambert, Augusta (17). The oligonucleotides for making the NLuc-mini-Gα_s_ E392K and L388R mutations were designed using New England Biolabs’ NEBaseChanger online primer design tool, as forward *5’-CAGGCAGTATAAGCTGCTCTAAC-3’*, reverse *5’-GTAAGTCGCCTACGTAGA-3’* for NLuc-mini-Gα_s_ E392K mutation and forward *5’-CGGATGCATCGCAGGCAGTAT-3’*, reverse *5’-GACAGCCCTGTAGTAAGTC-3’* for NLuc-mini-Gα_s_ L388R mutation. Both mutations were generated by PCR mutagenesis and the sequences were confirmed by DNA sequencing at Source Biosciences (Cambridge, UK)

Human embryonic kidney (HEK) 293T cells were maintained in Dulbecco’s Modified Eagle’s Medium (DMEM) media (ThermoFisher, UK), supplemented with 10% Heat Inactivated Foetal Bovine Serum (FBS) (Sigma-Aldrich) and 100 U/ml penicillin and 0.1 mg/ml streptomycin (ThermoFisher, UK). Cells were maintained at 37 °C with 5% CO2, in a humidified atmosphere and passaged routinely. For BRET experiments, HEK293T cells were seeded at a density of 700,000 cell/well in a 6-well plate and grown overnight. The next day, cells were transfected with Lipofectamine 2000 (ThermoFisher) following the manufacturer’s protocol. For BRET experiments examining mini-G recruitment to PTH1R, 50ng of NLuc-tagged mini-G was transfected alongside 500ng C-terminally SNAP-tagged PTH1R, whereas, for mini-G recruitment to plasma membrane, 50ng of NLuc-tagged mini-G was transfected alongside 500ng Venus-K-Ras, -Rab5, -Rab4, -Rab11a and 300ng PTH1R. After 24 hours, cells were re-seeded onto white 96-well white polystyrene Nunc microplates (Sigma) precoated with poly-D-lysine (PDL)-coated, at a density of 100,000 cells/well in a complete FluoroBrite phenol red-free DMEM medium supplemented with 4 mM L-glutamine and 5% FBS medium and grown overnight. The next day, SNAP-tagged transfected cells were labelled with 1μM BG-Cy5 in complete FluoroBrite phenol red-free DMEM medium (without antibiotic) for 1 hour at 37 °C. Cells were then washed three times with complete FluoroBrite phenol red-free DMEM medium, followed by 80μL Hank’s balanced salt solution (HBSS) containing 10mM HEPES and 10μM furimazine/NanoGlo Luciferase Assay Substrate (Promega). BRET measurements were performed at 37 °C using a PHERAstar Microplate Reader (BMG Labtech) with a dual-luminescence readout BRET plus filter (460-490 nm band-pass, 520-550 nm long-pass). The BRET signal was recorded for 4 baseline measurements before the addition of 20μL PTH(1-34), and measured for an additional hour. The corresponding BRET ratio was calculated as the ratio of light emission from YFP (520 nm) over NLuc (460 nm). Net BRET ratio was baseline-corrected with vehicle-treated cells and normalized to the baseline values.

### Gα_s_ activation analysis with TRUPATH

The pJG3.6-PTH1R construct was given to us by Dr. Simon Dowell (GSK, Stevenage, UK) and the β2-adrenoceptor (ADRB2) construct was gifted by Asuka Inoue (Tohoku University). The TRUPATH components including Gαs-Rluc8, Gβ3 and Gγ9-GFP2 were purchased as part of the TRUPATH biosensor kit from Addgene. The oligonucleotides for making the GαsE392K-Rluc8 and GαsL388R-Rluc8 mutations were designed using Agilent Technologies’ online primer design tool, as forward *5’-gagctattatagcagcttgtactgacgaaggtgca-3’*, reverse *5’-tgcaccttcgtcagtacaagctgctataatagctc-3’* for GαsE392K-Rluc8 and forward *5’-gctcgtactgacgacggtgcatgcgctga-3’* and reverse *5’-tcagcgcatgcaccgtcgtcagtacgagc-3’* for the GαsL388R-Rluc8 mutation. Both mutations were made using the QuikChange Lightning Site directed Mutagenesis Kit (Agilent Technologies) according to the manufacturer’s instructions and the sequences were confirmed by DNA sequencing at the Department of Biochemistry (University of Cambridge, UK). Peptide PTH(1-34) was purchased from Bachem (Bubendorf, Switzerland) and isoprenaline was purchased from Sigma (UK). Both were dissolved in dimethyl sulfoxide (DMSO) and stored as 1mM stock in -20°C.

HEK293T cells were maintained in Dulbecco’s Modified Eagle’s Medium (DMEM)/Hams F-12 nutrient mix (F12) GlutaMAXTM media (ThermoFisher, UK), supplemented with 10% Heat Inactivated Foetal Bovine Serum (FBS) (Sigma-Aldrich, Poole, Dorset, UK) and 1% antibiotic-antimycotic (AA) (Sigma, UK). Cells were maintained at 37 °C with 5% CO2, in a humidified atmosphere and passaged routinely. To perform the TRUPATH experiment, HEK293T cells were plated in a density of 1,500,000 cells/well in a 6-well plate and grown in complete DMEM /F-12 GlutaMAX™ overnight. The seeded cells were transfected using 25 kDa polyethylenimine (PEI, Polysciences Inc., Germany) at a 6:1 ratio of PEI to DNA, diluted in 150mM NaCl. The receptor, Gαs-RLuc8 WT/E392K/L388R, Gβ3, Gγ9-GFP2 and pcDNA3.1 were transfected together with a ratio of 1:1:1:1:1 using 400 ng per construct. 24 hours post transfection, cells were trypsinized and re-seeded onto white 96-well plates (Greiner, UK) precoated with poly-L-lysine (PLL)-coated, at a density of 50,000 cells/well in a complete DMEM/F12 medium and grown overnight. On the next day, the cell culture media was removed, followed by wells washed with Hank’s balanced salt solution (HBSS). 80μl assay buffer (1× HBSS with calcium and magnesium, supplemented with 20 mM HEPES and 0.1% BSA with pH adjusted to 7.4) were added to each well, together with 10μl of coelenterazine 400a (Nanolight technology, USA) to a final concentration of 5 μM. For PTH1R experiments, the plates were then incubated in the dark for 5 minutes followed by the addition of 10 μl PTH(1-34) (ranging from 0.01nM to 10μM). For ADRB2 experiments, the TRUPATH G protein activation assay was performed using 10μM isoprenaline. The BRET signals were recorded every 60 seconds for 15 minutes on a Mithras LB940 plate reader and the corresponding BRET ratio was calculated as the ratio of light emission from GFP2 (515 nm) over Rluc8 (400 nm). Net BRET ratio was baseline-corrected with DMSO response and the negative peak in area under the curve (AUC) analysis was used to generate the dose-response curves. The dose-response curves were fitted with the three-parameter logistic equation built in Prism 9.3.1 (Graphpad Prism, San Diego, CA) for determining the response potency (pEC50). The statistical significance was calculated using ordinary one-way ANOVA with a Dunnett’s multiple comparisons test built in Prism 9.3.1.

### cAMP accumulation assay

The HEK293ΔGNAS cell line was kindly gifted by Asuka Inoue. The pcDNA3.1-Gs-short construct was purchased from cDNA.org and the mutants GαsE392K and GαsL388R were generated and verified as described previously for the Gαs-RLuc8 mutants. HEK293ΔGNAS cells were maintained in DMEM/F12 GlutaMaxTM medium supplemented with 10% FBS and 1% AA. Cells were plated at a density of 300,000 cells/well in a 24-well plate and grown overnight prior to transfection. Transfection was performed with FugeneHD (Promega, UK) at a 3:1 ratio to DNA in accordance with the manufacturer’s instructions. PTH1R and GαsWT/GαsE392K/GαsL388R/pcDNA3.1 were transfected using 250ng per construct. After 48-hour transfection, cells were harvested and resuspended in the stimulation buffer (Phosphate buffer saline containing 0.1% BSA and 2.5mM isobutylmethylxanthine). The resuspended cells were then seeded at 1000 cells per well in 384-well white Optiplates (Perkin Elmer) and incubated with PTH(1-34) (ranging from 10μM to 10pM) or forskolin (Sigma, UK, ranging from 100μM to 100pM) for 1 hour at room temperature. The accumulated cAMP level was detected using the LANCE ultra cAMP detection kit on a Mithras LB 940 multimode microplate reader (Berthold Technologies). The response was normalized against the cAMP level produced from the stimulation of 100μM forskolin and fitted with the three-parameter logistics equation built in Prism 9.3.1 (Graphpad Prism, San Diego, CA).

### Systems pharmacology models of Gα_s_ activation and signaling

A mechanistic, kinetic model as presented in the reaction scheme in Fig. 3A was generated using the reactions and parameters lists available in tables S1 and S2. Using the law of mass action, a system of Ordinary Differential Equations (ODEs) was derived in COPASI 4.37 (Build 264) (32). The initial concentration of inactive receptor (Ri) and unbound heterotrimeric G protein (G) were both set to 415pM, as previously implemented (21). All receptor and G protein species were allowed to equilibrate for 10^6^ seconds before addition of ligand. The LSODA solver in COPASI (32) computationally solved our system of ODEs, yielding species’ concentrations over time.

To compare simulated G protein concentration with the TRUPATH experimental data, the following linear transformation was applied:

$$Simulated\;TRUPATH\;=\frac{[G_{tot}]-{\left[G_{tot}\right]}_{L}}{{\left[G_{tot}\right]}_{L}}\cdot TP_{Sys.Max\;}$$

The concentration of total heterotrimeric G protein ([G_tot_]) was normalized and baseline-corrected to the resting G protein concentration ([G_tot_]_L_), where [G_tot_]_L_ is [G_tot_] measured at the time of ligand addition. To obtain values directly comparable to the experimental data, this normalized concentration was multiplied by TP_Sys.Max_, a constant representing the theoretical maximum TRUPATH output in our system if all G protein was activated. To fit simulated results to TRUPATH data on the wild type Gαs, COPASI’s parameter estimation task was used (32). Optimization algorithms minimized the sum of squares (SoS) between experimental and simulated results at various ligand concentrations (1μM, 100nM, 10nM, 1nM). A final fit was found using a Genetic Algorithm with Stochastic Ranking with a SoS of 0.0733. To model experimental TRUPATH data for the L388R and E392K mutants, parameter space was scanned for parameters representing properties upstream of G protein signaling: K_G_, β, γ, K_GDA_, and K_GRA_. (table S2). Parameters of K_G+_ for the L388R mutant and K_GDA+_ for the E392K mutant (table s3) were selected based on agreement with mini-G experiments and close fitting to TRUPATH data, with a SoS between experimental and simulated data of 0.0956 for the L388R mutant and of 0.1041 for the E392K mutant.

To incorporate cAMP responses into the model, simplified, collective reactions for cAMP synthesis were appended (Fig. 3F, table S1). The initial concentration of AC was set to 4.15μM, while the initial concentration of ATP was set to 100μM. To examine the effects of theoretical rescue ligands in the model, model parameters representing properties of the ligand were scanned: K_L_, α and γ (table S2). Additionally, a Dual G protein model from the original mechanistic model (Fig. 5C and table S4) was derived by introducing an additional G protein at a concentration of 415pM. While Gα subunits of the two G proteins are distinct species, the Gβγ subunits from each G protein have identical properties. The final models have been made available in BioModels(33) and are accessible via the identifiers MODEL2306220001 and MODEL2306210001.

### Modelling and molecular dynamics of Gα_s_ – PTH1R complexes

In order to assess the structural impact of the mutations on G protein / PTH1R interactions we first modelled both L388R and E392K mutations into the available cryo-EM structure of PTH1R bound to the Gs protein and a PTH analogue (11) (PDB ID 6NBF). These mutationswere introduced using the Protein Builder function of the Chemical Computing Group’s Molecular Operating Environment (MOE) software (version 2022.02) and final structural representations were obtained with VMD1.9.4 (34).

To set up the molecular dynamics simulation systems further protein curation was carried out using the MOE software including: (i) reverting A118 to the WT G188, (ii) modelling of missing sidechains and (iii) assignment of protonation states. Afterwards, internal water molecules were added using the HOMOLWAT server (35). Then, the curated protein was embedded into a solvated and ionized (150 mM NaCl) lipid bilayer composed of POPC (1-palmitoyl-2-oleoylphosphatidylcholine) using the CHARMM GUI membrane builder (36). The E392K mutant system was generated following the same protocol using the CHARMM GUI membrane builder. To run the molecular dynamics simulations, we employed the ACEMD v3.3 software(37). The systems underwent an initial energy minimization consisting of 500 steps to mitigate steric clashes and optimize the initial structure. Subsequently, an equilibration step was performed in the NPT ensemble for a duration of 10 nanoseconds. During this phase, restraints were applied to the protein Cα atoms at a force constant of 1 kcal/mol, as well as to the remaining heavy atoms at a force constant of 0.1 kcal/mol. These restraints were gradually reduced until reaching 0 kcal/mol over the 10 nanoseconds. For the production phase, the systems were allowed to freely evolve for a total time of 1 microsecond in three replicates. The production runs were carried out using a timestep of 4 femtoseconds in the NVT ensemble, where the temperature was kept constant at 300 K. Simulation data has been deposited at the GPCRmd repository (38) and can be accessed via via IDs 1296 and 1297.

To compare the stability of interactions between PTH1R and the H5 of Gα_s_ we analyzed each simulation frame across the 3 independent wild type and E392K mutant MD replicates with VMD1.9.4 (34) to select all receptor residues found within 4Å of residues 382 to 392 in the Gα protein (see Supplementary Data 2 for a list of all interactions). We considered an interaction to pass our stability filter when it occurred in more than 75% of the analyzed frames. Per-residue secondary structure calculations over the simulation trajectories for the H5 of Gα_s_ in wild type and E392K mutant conditions were obtained via the Timeline plugin implemented in VMD1.9.4. Final heatmap plots of those receptor residues establishing stable interactions with wild type and mutated Gα_s_ were generated using RStudio 2022.07.2 and structural representations were obtained with VMD1.9.4(34).

## Supplementary Material

Data file S1

Data file S2

Supplementry materials

## Data Availability

The structures of 135 receptors in complex with Gα_s_ that were used for comparative structural analysis (PDB IDs 6P9X, 7F4I, 7VUI, 7L1U, 6VN7, 7JOZ, 6WI9, 7CKY, 6P9Y, 7TZF, 7LCI, 7KI1, 7VUJ, 6XOX, 6VCB, 7CX3, 7RMG, 7CKW, 7DUR, 7E14, 7RG9, 7S3I, 7DHR, 7TYX, 6LI3, 7V35, 7BB6, 6ORV, 7D3S, 7F53, 7LJC, 7JVP, 7F58, 7XTC, 7PIV, 7CFN, 7TYH, 7P02, 7WU3, 6E67, 7VAB, 6X1A, 6M1H, 7TYY, 7RMH, 7TYF, 7DUQ, 7PIU, 7F54, 7TYI, 7KI0, 7D7M, 7EVM, 7F4H, 7BB7, 7XT8, 6X18, 7CZ5, 5UZ7, 7BZ2, 6NIY, 7VBI, 7RGP, 7RBT, 7F4D, 5VAI, 6PB0, 7RA3, 7CX2, 6PB1, 6LPB, 7TYW, 6WPW, 7RTB, 7JV5, 7LLL, 7F16, 7DW9, 7XTB, 7TYO, 6LMK, 6WHC, 6WZG, 6GDG, 7DHI, 7CKZ, 7CKX, 7VUH, 6B3J, 7EVW, 7BW0, 7CRH, 7LJD, 7F55, 7L1V, 6M1I, 7AUE, 6E3Y, 7S1M, 5G53, 7DH5, 7LLY, 7RMI, 7TYL, 6X19, 6UUN, 7EZK, 6UUS, 7WUJ, 7MBX, 7VBH, 7CX4, 7CFM, 6NI3, 7F4F, 7V9M, 7WU2, 7KH0, 6UVA, 7TYN, 7C2E, 7JVQ, 6NBF, 7JJO, 7FIG, 7D68, 6NBI, 7DTY, 7FIY, 7FII, 3SN6, 7FIN, 7FIH, 7FIM, 6NBH) were downloaded from the Protein Data Bank (https://rcsb.org/), as well as the starting structure used to model Gα_s_ mutation effects and for MD simulations (PDB ID 6NBF). Information on primary receptor couplings as annotated in the Guide to Pharmacology was downloaded from the G protein couplings resource available through the GPCR database (https://gproteindb.org/signprot/couplings). Transcriptomics information on receptor expression in the kidney was obtained from the Kidney Transcriptomics Data resource from the Epithelial Systems Biology Laboratory at NHLBI, NIH, Bethesda, MD (https://esbl.nhlbi.nih.gov/Databases/KSBP2/Targets/TranscriptomicData.html). Venus-tagged membrane markers for mini-Gα_s_ characterization were obtained via a Material Transfer Agreement with the University of Western Australia. MD simulation data has been deposited at the GPCRmd repository and can be found via IDs 1296 and 1297. Systems pharmacology models have been deposited in the BioModels database (https://www.ebi.ac.uk/biomodels/) and can be accessed via the identifiers MODEL2306220001 and MODEL2306210001. All other data needed to evaluate the conclusions in the paper are present in the paper or the Supplementary Materials.
