# Supplementary material for: Loss-of-function Gαs rare disease variants exert mutation-specific effects on GPCR signaling
Source: Sci Signal. Author manuscript; Available in PMC 2025 Jun 9. (PMC7617749; doi:10.1126/scisignal.ado7543)
Supplement: Supplementry materials [file EMS205175-supplement-Supplementry_materials.pdf]

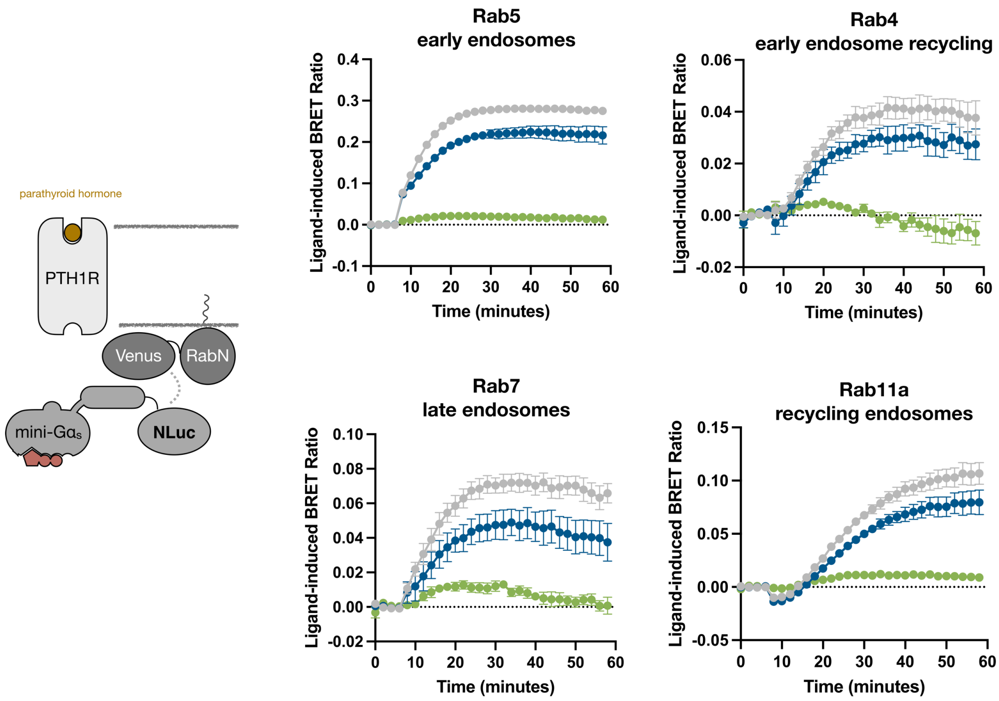

Fig. S1. Monitoring ligand-induced mini-Gα_s recruitment to endosomes. BRET assays used to measure mini-Gα_s (WT, E392K, and L388R) recruitment to endosomal markers Rab5, Rab4, Rab7, and Rab11a following stimulation with 1µM PTH (1-34). The BRET ratio was calculated as acceptor/donor wavelength and baseline-corrected with the value obtained with vehicle control. All values are represented as mean ± SEM, with n=3 independent experimental repeats performed in duplicate.

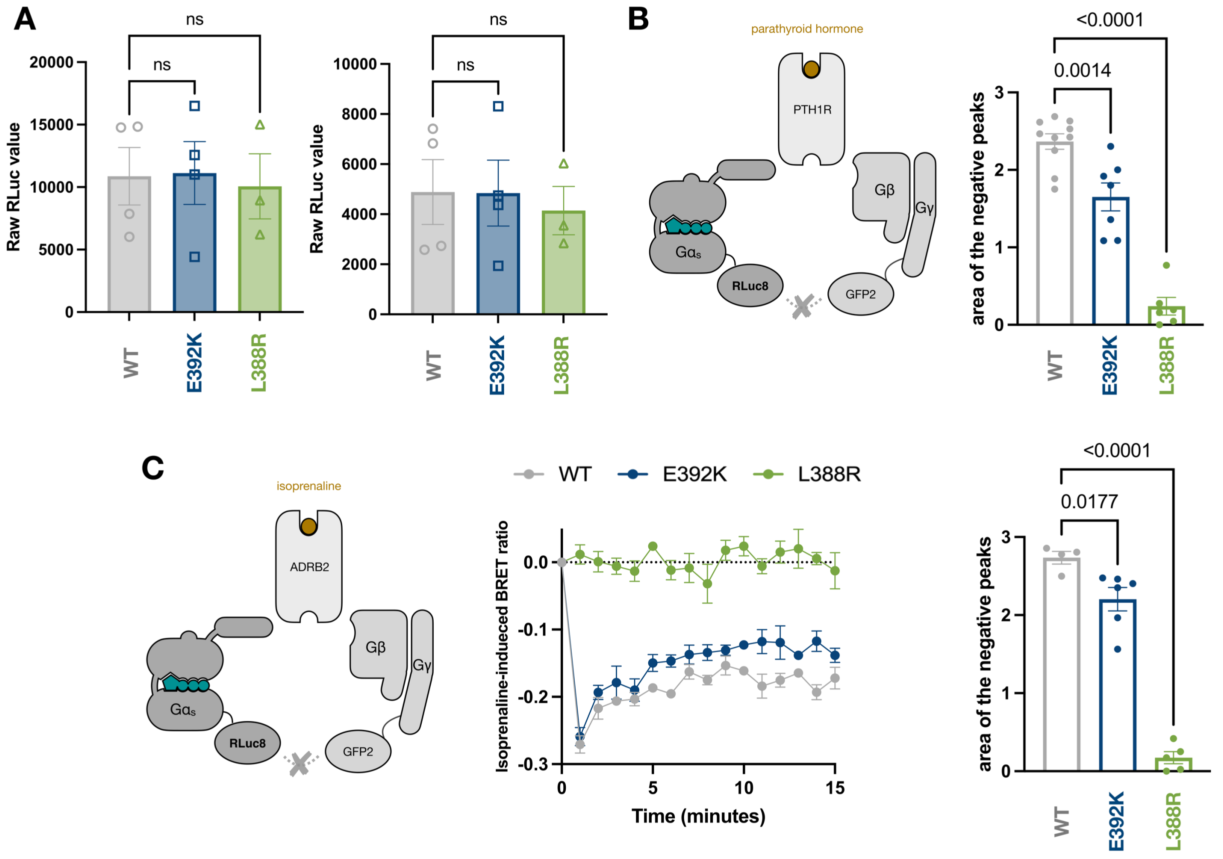

Fig. S2. Activation of Gα protein variants by different $G\alpha_s$-coupled receptors. (**A**) Raw RLuc values at t = 1 minute (left) and t = 15 minutes (right). All values are represented as mean ± SEM, with at least 3 independent experimental repeats conducted in duplicates. One-way AVOVA with Dunnett's multiple comparison test was used to determine the statistical significance of the difference of the RLuc values between WT, E392K, or L388R $G\alpha_s$-RLuc8. (**B**) Schematic representation of PTH1R-induced G protein activation measured in the TRUPATH assay and area of the negative peaks of 15min baseline-corrected BRET response induced by 1μM PTH(1-34) for WT, L388R, and E392K $G\alpha_s$. n = 3 independent experimental repeats conducted in duplicate. (**C**) Schematic representation of β_2 adrenergic receptor (ADRB2)-induced G protein activation measured in the TRUPATH assay. The middle panel shows dissociation of heterotrimeric G_s containing WT, L388R, or E392K $G\alpha_s$, as determined using the TRUPATH G protein activation assay following stimulation of ADRB2 using 10μM isoprenaline. The BRET ratio was calculated as GFP2/RLuc8 and baseline-corrected with the value obtained with DMSO as control. The right panel shows the area of the negative peaks of 15min baseline-corrected BRET response as in (B). All values are represented as mean ± SEM, with n=3 independent experimental repeats conducted in duplicate. One-way AVOVA with a Dunnett's multiple comparisons test was performed to compare the statistical significance of the differences in G protein activation level between $G\alpha_s$ WT and $G\alpha_s$ E392K or L388R.

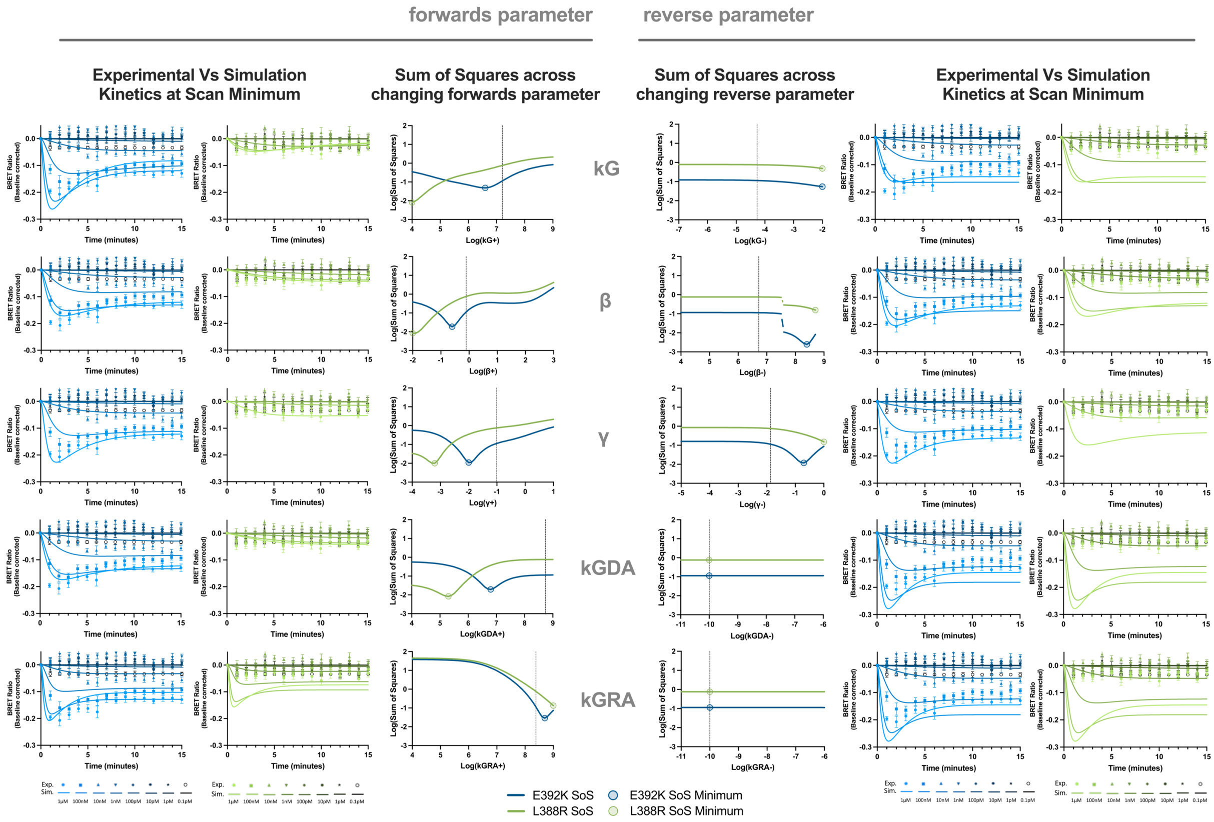

Fig. S3. Mutant parameter space exploration. Time-course values for the TRUPATH G protein activation assay following 15-minute stimulation of PTH1R using 1μM – 0.1pM PTH(1-34) represented as points, with curves corresponding to simulations of the E392K or L388R mutant using parameter values that minimize the sum of squares presented in the adjoining line plot. The line plot represents the sum of squares (SoS) values between simulated and experimental points for the E392K (blue) or L388R (green) mutant. The SoS values were calculated following the corresponding change to the given parameter (x-axis). The vertical dashed line represents the starting (WT) parameter. Circles correspond to the lowest SoS value for each parameter scan. n = 3 independent experimental repeats conducted in duplicate.

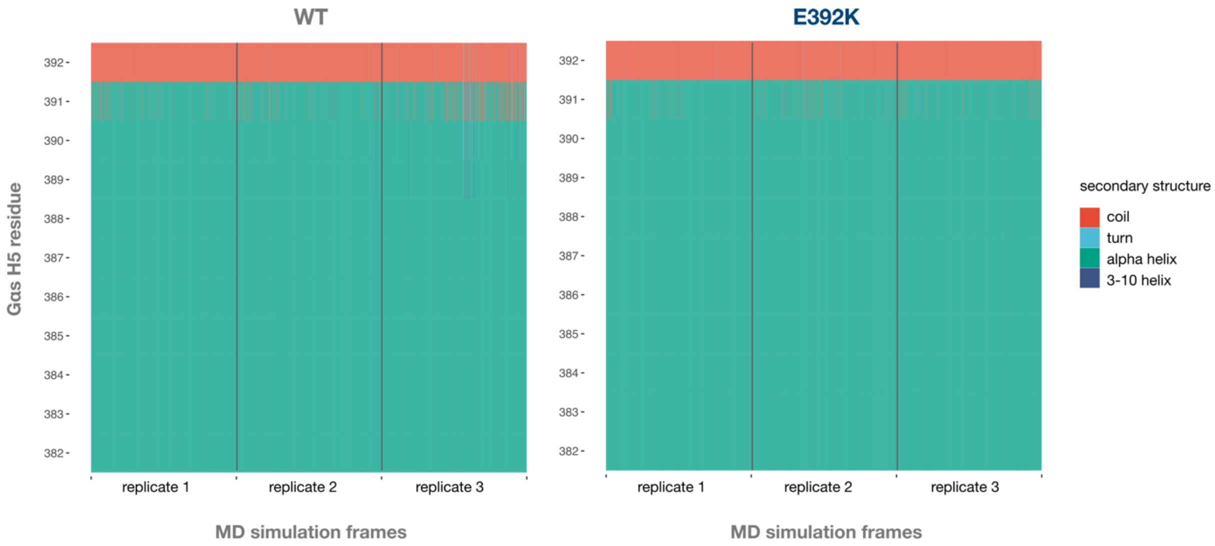

Fig. S4. Gα_s H5 secondary structure across MD simulation trajectories. Heatmap representation of residue-specific secondary structure assignments per simulation frame across 3 independent 1 μs replicates. Values were calculated with the VMD1.9.4 Timeline plugin and correspond to residues 382 to 392 of the helix 5 (H5) of Gα_s from simulations with a wild type (WT) and a mutated (E392K) G protein.

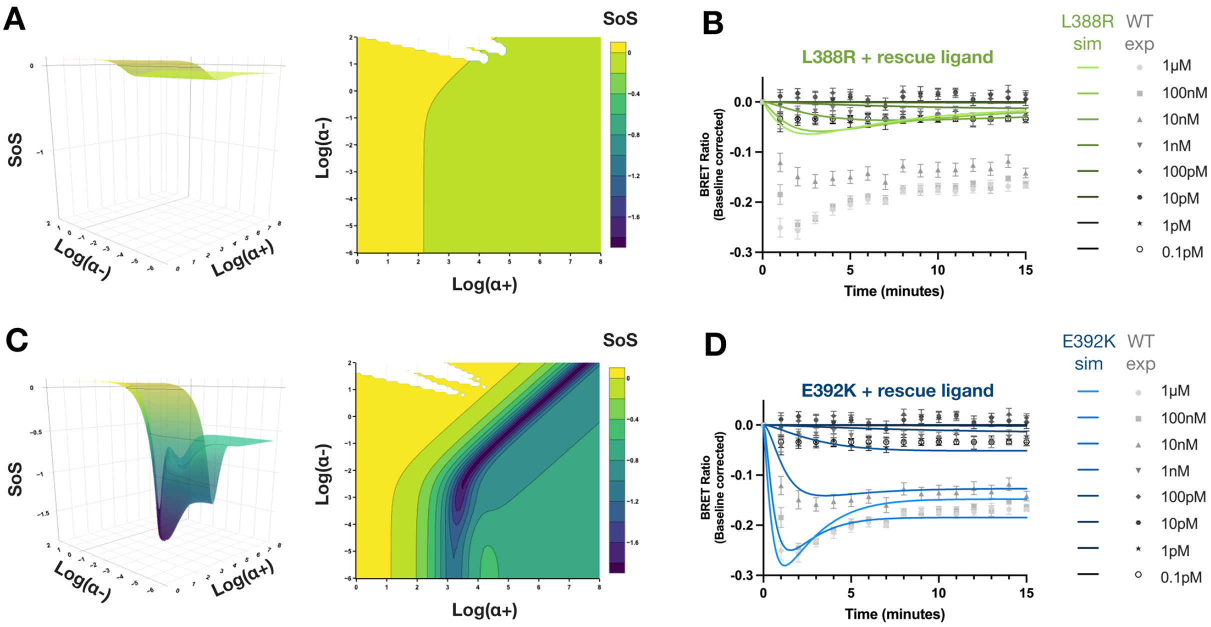

Fig. S5. Rescue ligand parameter space exploration. (**A**) Surface representation of sum of squares (SoS) values corresponding to the fit between simulations of the L388R mutant and WT experimental values upon altering the forward and backward ligand-receptor cooperativity factor (α+ and α-). (**B**) Time-course values for the TRUPATH G protein activation assay following 15-minute stimulation of the PTH1R using 1μM – 0.1pM PTH(1-34) for WT represented as points, with curves corresponding to simulations of the L388R mutant stimulated by an ideal ligand with altered cooperativity factor values (α+ and α-) corresponding to the SoS minimum. (**C**) SoS values corresponding to the fit between simulations of the E392K mutant and WT experimental values upon altering the forward and backward ligand-receptor cooperativity factor (α+ and α-). (**D**) Time-course values for the TRUPATH G protein activation assay following 15-minute stimulation of the PTH1R using 1μM – 0.1pM PTH(1-34) for WT represented as points, with curves corresponding to simulations of the E392K mutant stimulated by an ideal ligand with altered cooperativity factor values (α+ and α-) corresponding to the SoS minimum. n = 3 independent experimental repeats conducted in duplicate.

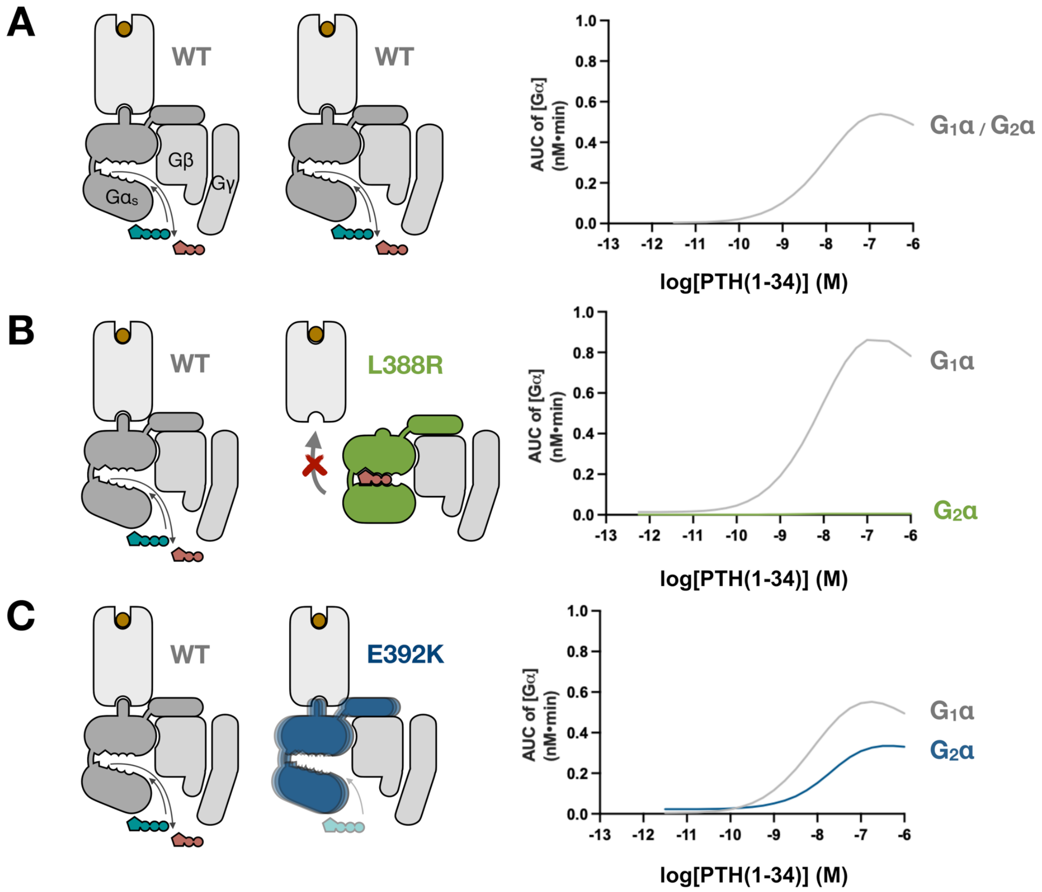

Fig. S6. Activation of individual G proteins in the dual G protein model. Simulated curves for total free Gα accumulation corresponding to G_1 and G_2 proteins in the dual G protein model (Fig. 5C). (**A to C**) Total free $G\alpha_s$ accumulation in the presence of two WT $G\alpha_s$ proteins (A), one WT $G\alpha_s$ and one L388R mutant $G\alpha_s$ (B), or one WT $G\alpha_s$ and one E392K mutant $G\alpha_s$.

Table S1. Reaction list for the G protein activation model.

	Reaction	Constituent Parameters	Value	Units
1	L + Ri ⇌ LRa	k_{L+}<br>k_{L-}	1.36E+07<br>3.61E+00	$M^{-1}\ s^{-1}$<br>s^{-1}
2	Ri ⇌ Ra	k_{act+}<br>k_{act-}	3.84E-06<br>2.54E+01	s^{-1}<br>s^{-1}
3	Ri + G ⇌ RiG	k_{G+}<br>k_{G-}	1.60E+07<br>5.33E-05	$M^{-1}\ s^{-1}$<br>s^{-1}
4	LRi ⇌ LRa	$k_{act+} \cdot \alpha_{+}$<br>$k_{act-} \cdot \alpha_{-}$	9.92E-03<br>2.59E-01	s^{-1}<br>s^{-1}
5	LRi + G ⇌ LRiG	$k_{G+} \cdot \gamma_{+}$<br>$k_{G-} \cdot \gamma_{-}$	1.58E+06<br>7.17E-07	$M^{-1}\ s^{-1}$<br>s^{-1}
6	Ra + G ⇌ RaG	$k_{G+} \cdot \beta_{+}$<br>$k_{G-} \cdot \beta_{-}$	1.26E+07<br>2.79E+02	$M^{-1}\ s^{-1}$<br>s^{-1}
7	L + Ra ⇌ LRa	$k_{L+} \cdot \alpha_{+}$<br>$k_{L-} \cdot \alpha_{-}$	3.51E+10<br>3.68E-02	$M^{-1}\ s^{-1}$<br>s^{-1}
8	L + RiG ⇌ LRiG	$k_{L+} \cdot \gamma_{+}$<br>$k_{L-} \cdot \gamma_{-}$	1.34E+06<br>4.86E-02	$M^{-1}\ s^{-1}$<br>s^{-1}
9	RiG ⇌ RaG	$k_{act+} \cdot \beta_{+}$<br>$k_{act-} \cdot \beta_{-}$	3.02E-06<br>1.33E+08	s^{-1}<br>s^{-1}
10	LRa + G ⇌ LRaG	$k_{G+} \cdot \beta_{+} \cdot \gamma_{+} \cdot \delta_{+}$<br>$k_{G-} \cdot \beta_{-} \cdot \gamma_{-} \cdot \delta_{-}$	1.24E+06<br>3.76E+00	$M^{-1}\ s^{-1}$<br>s^{-1}
11	L + RaG ⇌ LRaG	$k_{L+} \cdot \alpha_{+} \cdot \gamma_{+} \cdot \delta_{+}$<br>$k_{L-} \cdot \alpha_{-} \cdot \gamma_{-} \cdot \delta_{-}$	3.46E+09<br>4.96E-04	$M^{-1}\ s^{-1}$<br>s^{-1}
12	LRiG ⇌ LRaG	$k_{act+} \cdot \alpha_{+} \cdot \beta_{+} \cdot \delta_{+}$<br>$k_{act-} \cdot \alpha_{-} \cdot \beta_{-} \cdot \delta_{-}$	7.79E-03<br>1.36E+06	s^{-1}<br>s^{-1}
13	RaG ⇌ Ra + GαGTP + Gβγ	k_{GDA+}<br>k_{GDA-}	5.49E+08<br>1.00E-10	s^{-1}<br>$M^{-2}\ s^{-1}$
14	LRaG ⇌ LRa + GαGTP + Gβγ	$k_{GDA+} \cdot \gamma_{-}$<br>$k_{GDA-} \cdot \gamma_{+}$	5.41E+07<br>1.35E-12	s^{-1}<br>$M^{-2}\ s^{-1}$
15	GαGTP ⇌ GαGDP	k_{hyd+}<br>k_{hyd-}	5.15E-01<br>2.00E-05	s^{-1}<br>s^{-1}
16	GαGDP + Gβγ ⇌ G	k_{GRA+}<br>k_{GRA-}	2.42E+08<br>1.00E-10	$M^{-1}\ s^{-1}$<br>s^{-1}
17	GαGTP + AC + ATP ⇌ GαGTPAC + cAMP	k_{syn+}<br>k_{syn-}	1.00E+05<br>1.00E-20	$M^{-2}\ s^{-1}$<br>$M^{-1}\ s^{-1}$
18	cAMP ⇌ ATP	k_{deg+}<br>k_{deg-}	1.00E-01<br>1.00E-20	s^{-1}<br>s^{-1}
19	GαGTPAC ⇌ GαGDP + AC	k_{ACU+}<br>k_{ACU-}	1.00E-03<br>1.00E-20	s^{-1}<br>$M^{-1}\ s^{-1}$

Table S2. Description of reaction parameters for the G protein activation model.

Label	Description	Value	Units
k_{L+}	Ligand binding rate	1.36E+07	M^{-1} s^{-1}
k_{L-}	Ligand unbinding rate	3.61E+00	s^{-1}
k_{act+}	Receptor activation rate	3.84E-06	s^{-1}
k_{act-}	Receptor deactivation rate	2.54E+01	s^{-1}
k_{G+}	G protein binding rate	1.60E+07	M^{-1} s^{-1}
k_{G-}	G protein unbinding rate	5.33E-05	s^{-1}
α_{+}	Forward cooperativity factor for ligand bound receptor activation	2.58E+03	-
α_{-}	Backwards cooperativity factor for ligand bound receptor activation	1.02E-02	-
β_{+}	Forward cooperativity factor for G protein-bound receptor activation	7.85E-01	-
β_{-}	Backwards cooperativity factor for G protein-bound receptor activation	5.24E+06	-
γ_{+}	Forward cooperativity factor for ligand binding a G protein-bound receptor	9.86E-02	-
γ_{-}	Backwards cooperativity factor for ligand binding a G protein-bound receptor	1.35E-02	-
δ_{+}	Forward cooperativity factor for ligand bound, G protein-bound receptor activation	1.00E+00	-
δ_{-}	Backwards cooperativity factor for ligand bound, G protein-bound receptor activation	1.00E+00	-
k_{GDA+}	G protein dissociation rate from active, G protein-bound receptor (possibly ligand-bound)	5.49E+08	s^{-1}
k_{GDA-}	Reformation of active, G protein-bound receptor (possibly ligand-bound)	1.00E-10	M^{-2} s^{-1}
k_{GRA+}	Heterotrimeric G protein reassociation rate	2.42E+08	M^{-1} s^{-1}
k_{GRA-}	Heterotrimeric G protein spontaneous dissociation rate	1.00E-10	s^{-1}
k_{hyd+}	Rate of hydrolysis of GαGTP	5.15E-01	s^{-1}
k_{hyd-}	Spontaneous exchange rate of GDP for GTP	2.00E-05	s^{-1}
k_{syn+}	Collective rate of GαGTP binding to AC and of cAMP synthesis	1.00E+05	M^{-2} s^{-1}
k_{syn-}	Collective reverse rate of GαGTP binding to AC and of cAMP synthesis	1.00E-20	M^{-1} s^{-1}
k_{deg+}	Collective rate of cAMP degradation	1.00E-01	s^{-1}
k_{deg-}	Collective reverse rate of cAMP degradation	1.00E-20	s^{-1}
k_{ACU+}	Collective rate of GαGTP unbinding from AC and GTP hydrolysis to GDP	1.00E-03	s^{-1}
k_{ACU-}	Collective reverse rate of GαGTP unbinding from AC and GTP hydrolysis to GDP	1.00E-20	M^{-1} s^{-1}

Table S3. Parameter changes to simulate L388R and E392K mutants in the G protein activation model.

Parameter	E392K		L388R		Units
	New Value	Fold Reduction compared to WT	New Value	Fold Reduction compared to WT	
k_{L+}	-	-	-	-	M^{-1} s^{-1}
k_{L-}	-	-	-	-	s^{-1}
k_{act+}	-	-	-	-	s^{-1}
k_{act-}	-	-	-	-	s^{-1}
k_{G+}	-	-	1.60E+04	1000	M^{-1} s^{-1}
k_{G-}	-	-	-	-	s^{-1}
α_+	-	-	-	-	-
α_-	-	-	-	-	-
γ_+	-	-	-	-	-
γ_-	-	-	-	-	-
β_+	-	-	-	-	-
β_-	-	-	-	-	-
δ_+	-	-	-	-	-
δ_-	-	-	-	-	-
k_{GDA+}	7.67E+06	72	-	-	s^{-1}
k_{GDA-}	-	-	-	-	M^{-2} s^{-1}
k_{GRA+}	-	-	-	-	M^{-1} s^{-1}
k_{GRA-}	-	-	-	-	s^{-1}
k_{hyd+}	-	-	-	-	s^{-1}
k_{hyd-}	-	-	-	-	s^{-1}

Table S4. Reaction list for the dual G protein activation model.

	Reaction	Constituent Parameters	Value	Units
1	L + Ri ⇌ LRa	k_{L+}<br>k_{L-}	1.36E+07<br>3.61E+00	$M^{-1}\ s^{-1}$<br>s^{-1}
2	Ri ⇌ Ra	k_{act+}<br>k_{act-}	3.84E-06<br>2.54E+01	s^{-1}<br>s^{-1}
3	LRi ⇌ LRa	$k_{act+} \cdot \alpha_{+}$<br>$k_{act-} \cdot \alpha_{-}$	9.91E-03<br>2.59E-01	s^{-1}<br>s^{-1}
4	L + Ra ⇌ LRa	$k_{L+} \cdot \alpha_{+}$<br>$k_{L-} \cdot \alpha_{-}$	3.51E+10<br>3.68E-02	$M^{-1}\ s^{-1}$<br>s^{-1}
5	Ri + G1 ⇌ RiG1	k_{G1+}<br>k_{G1-}	1.60E+07<br>5.33E-05	$M^{-1}\ s^{-1}$<br>s^{-1}
6	LRi + G1 ⇌ LRiG1	$k_{G1+} \cdot \gamma_{1+}$<br>$k_{G1-} \cdot \gamma_{1-}$	1.58E+06<br>7.20E-07	$M^{-1}\ s^{-1}$<br>s^{-1}
7	Ra + G1 ⇌ RaG1	$k_{G1+} \cdot \beta_{1+}$<br>$k_{G1-} \cdot \beta_{1-}$	1.26E+07<br>2.79E+02	$M^{-1}\ s^{-1}$<br>s^{-1}
8	LRa + G1 ⇌ LRaG1	$k_{G1+} \cdot \beta_{1+} \cdot \gamma_{1+}$<br>$k_{G1-} \cdot \beta_{1-} \cdot \gamma_{1-}$	1.24E+06<br>3.77E+00	$M^{-1}\ s^{-1}$<br>s^{-1}
9	Ri + G2 ⇌ RiG2	k_{G2+}<br>k_{G2-}	1.60E+07<br>5.33E-05	$M^{-1}\ s^{-1}$<br>s^{-1}
10	LRi + G2 ⇌ LRiG2	$k_{G2+} \cdot \gamma_{2+}$<br>$k_{G2-} \cdot \gamma_{2-}$	1.58E+06<br>7.20E-07	$M^{-1}\ s^{-1}$<br>s^{-1}
11	Ra + G2 ⇌ RaG2	$k_{G2+} \cdot \beta_{2+}$<br>$k_{G2-} \cdot \beta_{2-}$	1.26E+07<br>2.79E+02	$M^{-1}\ s^{-1}$<br>s^{-1}
12	LRa + G2 ⇌ LRaG2	$k_{G2+} \cdot \beta_{2+} \cdot \gamma_{2+}$<br>$k_{G2-} \cdot \beta_{2-} \cdot \gamma_{2-}$	1.24E+06<br>3.77E+00	$M^{-1}\ s^{-1}$<br>s^{-1}
13	L + RiG1 ⇌ LRiG1	$k_{L+} \cdot \gamma_{1+}$<br>$k_{L-} \cdot \gamma_{1-}$	1.34E+06<br>4.87E-02	$M^{-1}\ s^{-1}$<br>s^{-1}
14	RiG1 ⇌ RaG1	$k_{act+} \cdot \beta_{1+}$<br>$k_{act-} \cdot \beta_{1-}$	3.01E-06<br>1.33E+08	s^{-1}<br>s^{-1}
15	LRiG1 ⇌ LRaG1	$k_{act+} \cdot \alpha_{+} \cdot \beta_{1+}$<br>$k_{act-} \cdot \alpha_{-} \cdot \beta_{1-}$	7.78E-03<br>1.36E+06	s^{-1}<br>s^{-1}
16	L + RaG1 ⇌ LRaG1	$k_{L+} \cdot \alpha_{+} \cdot \gamma_{1+}$<br>$k_{L-} \cdot \alpha_{-} \cdot \gamma_{1-}$	3.46E+09<br>4.97E-04	$M^{-1}\ s^{-1}$<br>s^{-1}
17	L + RiG2 ⇌ LRiG2	$k_{L+} \cdot \gamma_{2+}$<br>$k_{L-} \cdot \gamma_{2-}$	1.34E+06<br>4.87E-02	$M^{-1}\ s^{-1}$<br>s^{-1}
18	RiG2 ⇌ RaG2	$k_{act+} \cdot \beta_{2+}$<br>$k_{act-} \cdot \beta_{2-}$	3.01E-06<br>1.33E+08	s^{-1}<br>s^{-1}
19	LRiG2 ⇌ LRaG2	$k_{act+} \cdot \alpha_{+} \cdot \beta_{2+}$<br>$k_{act-} \cdot \alpha_{-} \cdot \beta_{2-}$	7.78E-03<br>1.36E+06	s^{-1}<br>s^{-1}

20	L + RaG2 ⇌ LRaG2	$k_{L+} \cdot \alpha_{+} \cdot \gamma_{2+}$<br>$k_{L-} \cdot \alpha_{-} \cdot \gamma_{2-}$	3.46E+09<br>4.97E-04	$M^{-1}\ s^{-1}$<br>s^{-1}
21	RaG1 ⇌ Ra + G1αGTP + Gβγ	k_{GDA1+}<br>k_{GDA1-}	5.49E+08<br>1.00E-10	s^{-1}<br>$M^{-2}\ s^{-1}$
22	RaG2 ⇌ Ra + G2αGTP + Gβγ	k_{GDA2+}<br>k_{GDA2-}	5.49E+08<br>1.00E-10	s^{-1}<br>$M^{-2}\ s^{-1}$
23	LRaG1 ⇌ LRa + G1αGTP + Gβγ	$k_{GDA1+} \cdot \gamma_{1-}$<br>$k_{GDA1-} \cdot \gamma_{1+}$	5.41E+07<br>1.35E-12	s^{-1}<br>$M^{-2}\ s^{-1}$
24	LRaG2 ⇌ LRa + G2αGTP + Gβγ	$k_{GDA2+} \cdot \gamma_{2-}$<br>$k_{GDA2-} \cdot \gamma_{2+}$	5.41E+07<br>1.35E-12	s^{-1}<br>$M^{-2}\ s^{-1}$
25	G1αGTP ⇌ G1αGDP	k_{hyd1+}<br>k_{hyd1-}	5.15E-01<br>2.00E-05	s^{-1}<br>s^{-1}
26	G2αGTP ⇌ G2αGDP	k_{hyd2+}<br>k_{hyd2-}	5.15E-01<br>2.00E-05	s^{-1}<br>s^{-1}
27	G1αGDP + Gβγ ⇌ G1	k_{GRA1+}<br>k_{GRA1-}	2.42E+08<br>1.00E-10	$M^{-1}\ s^{-1}$<br>s^{-1}
28	G2αGDP + Gβγ ⇌ G2	k_{GRA2+}<br>k_{GRA2-}	2.42E+08<br>1.00E-10	$M^{-1}\ s^{-1}$<br>s^{-1}

Data file S1. Gα_s-receptor protein contact analysis.

This Excel file lists GPCR / Gα_s contacts measured across 135 experimentally solved 3D structures. Residues are annotated using the G protein common numbering (CGN) scheme for Gα_s and GPCRdb generic numbering for receptors.

Data file S2. Contact frequencies between H5 in Gα_s and the PTH1R across MD trajectories.

This Excel file contains data on how many times a PTH1R residue contacted the WT or mutant (E392K) Gα_s across 3 different MD simulation replicates measured as the number of frames in which the residue was found within 4Å of residues 382 to 392 in the H5 of Gα_s.
